# Temporal and Quantitative Transcriptomic Differences Define Sexual Dimorphism in Murine Postnatal Bone Aging

**DOI:** 10.1002/jbm4.10579

**Published:** 2021-12-10

**Authors:** Darlene Lu, Serkalem Demissie, Nina B Horowitz, Adam C Gower, Marc E Lenburg, Yuriy O Alekseyev, Amira I Hussein, Beth Bragdon, Yu Liu, Dana Daukss, Jack M Page, Micheal Z Webster, Jennifer J Schlezinger, Elise F Morgan, Louis C Gerstenfeld

**Affiliations:** ^1^ Department of Biostatistics Boston University School of Public Health Boston MA USA; ^2^ Department of Orthopaedic Surgery Boston University School of Medicine Boston MA USA; ^3^ Department of Medicine, Section of Computational Biomedicine Boston University School of Medicine Boston MA USA; ^4^ Department of Pathology and Laboratory Medicine Boston University School of Medicine Boston MA USA; ^5^ Department of Mechanical Engineering Boston University Boston MA USA; ^6^ Department of Environmental Health Boston University School of Public Health Boston MA USA

**Keywords:** BONE AGING, SEX‐SPECIFIC, TEMPORAL TRANSCRIPTOMIC CLUSTER ANALYSIS

## Abstract

Time is a central element of the sexual dimorphic patterns of development, pathology, and aging of the skeleton. Because the transcriptome is a representation of the phenome, we hypothesized that both sex and sex‐specific temporal, transcriptomic differences in bone tissues over an 18‐month period would be informative to the underlying molecular processes that lead to postnatal sexual dimorphism. Regardless of age, sex‐associated changes of the whole bone transcriptomes were primarily associated not only with bone but also vascular and connective tissue ontologies. A pattern‐based approach used to screen the entire Gene Expression Omnibus (GEO) database against those that were sex‐specific in bone identified two coordinately regulated gene sets: one related to high phosphate–induced aortic calcification and one induced by mechanical stimulation in bone. Temporal clustering of the transcriptome identified two skeletal tissue‐associated, sex‐specific patterns of gene expression. One set of genes, associated with skeletal patterning and morphology, showed peak expression earlier in females. The second set of genes, associated with coupled remodeling, had quantitatively higher expression in females and exhibited a broad peak between 3 to 12 months, concurrent with the animals' reproductive period. Results of phenome‐level structural assessments of the tibia and vertebrae, and in vivo and in vitro analysis of cells having osteogenic potential, were consistent with the existence of functionally unique, skeletogenic cell populations that are separately responsible for appositional growth and intramedullary functions. These data suggest that skeletal sexual dimorphism arises through sex‐specific, temporally different processes controlling morphometric growth and later coupled remodeling of the skeleton during the reproductive period of the animal. © 2021 The Authors. *JBMR Plus* published by Wiley Periodicals LLC on behalf of American Society for Bone and Mineral Research.

## Introduction

Most mammals exhibit skeletal sexual dimorphism, with males commonly showing larger structural sizes of their skeletal elements and greater overall bone mass compared with females.^(^ ^1^, ^2^, ^3^, ^4^, ^5^, ^6^ ^)^ Differences in sex hormones, responsiveness to mechanical loading,^(^ ^7^ ^)^ and the insulin‐like growth factor axis^(^ ^8^ ^)^ have each been mechanistically linked to the sexual dimorphism of skeletal tissue development. Although both males and females lose bone mass with aging, bone loss in humans^(^ ^9^, ^10^ ^)^ and other mammals has been shown to greatly accelerate in females when systemic levels of estrogen decrease at menopause or after ovariectomy^(^ ^11^, ^12^ ^)^ or after genetic deletion of the estrogen receptor.^(^ ^13^ ^)^ The loss of estrogen increases bone turnover^(^ ^12^ ^)^ and has molecular effects on the interactions among the three cell types in bone (osteoclasts, osteoblasts, and osteocytes) that regulate bone remodeling.^(^ ^9^, ^13^ ^)^ There are also intrinsic sex‐associated differences in postnatal skeletal morphogenesis associated with earlier sexual maturation and earlier cessation in overall skeletal growth in females, which itself leads to a smaller skeletal mass in females compared with males.^(^ ^14^, ^15^ ^)^ Finally, emerging data suggest that there are immense sex‐specific differences in the coupled remodeling of the skeleton during the temporal period of female fecundity.^(^ ^16^ ^)^ Thus, the lower baseline skeletal mass of women, in combination with the increase in bone loss in the menopausal period, puts women at greater risk for osteoporotic fractures as they age.^(^ ^17^, ^18^ ^)^

Time is a central element to the development, pathology, and aging of the skeleton. Because the transcriptome is a representation of the phenome, we hypothesized that characterization of sex‐specific, temporal transcriptomic differences would be informative to understanding the underlying biological, molecular, and cellular processes that lead to postnatal sexual dimorphism in skeletal development, homeostasis, and aging. A series of bioinformatics approaches were used to analyze the sex‐associated changes of the bone transcriptome in male and female animals over an 18‐month period of postnatal life. We identified genes that showed differential quantitative sex‐specific expression regardless of animal age, as well as those whose temporal patterns of expression over the life span differed between the sexes. We then examined the correspondence between these gene ontologies and sex‐specific bone phenotypes, at both tissue and cellular levels.

## Materials and Methods

### Animals

**Fig 1:**
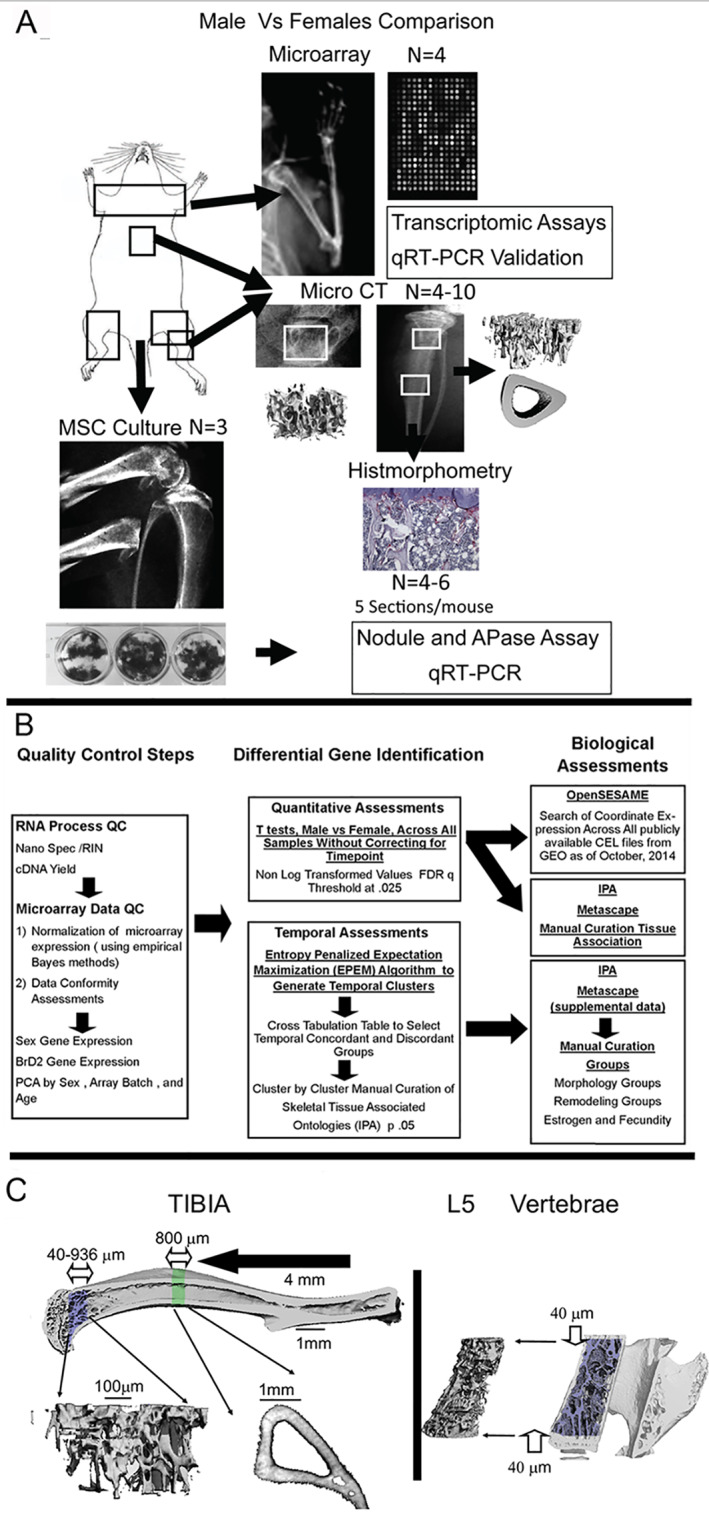
Schematic summary of work flow of the phenomic and transcriptomic analysis of experimental methods applied in this study. (*A*) Graphical scheme of the work flow of the primary animal group of 68 mice used for these analysis. All assays shown were performed in the same mice using different bones. All transcriptomic analysis was done in paired humeri from individual mice. All cell cultures were prepared from pooled marrow cells prepared from both femurs and the left tibia from one mouse. All microCT were performed on the right tibia and L_5_ and histomorphometry was sequentially done on the right tibia after microCT. Validation qRT‐PCR was carried out using total RNAs from the same samples used for microarray. In some cases, additional bones from a given age and sex group were included to increase the microCT group sizes to reach statistical significance. MicroCT was the only assay in which this was carried out. *N* values as denoted in the figure represent individual animal numbers within the assay group. (*B*) Flow diagram of the steps of the array analysis. The analysis proceeded from quality control steps (RNA QC and MicroArray QC), differential gene expression (quantitative and temporal), biological assessments (openSESAME, IPA Metascape, and manual annotation). (*C*) Graphical scheme of the analytical steps of the microCT analysis. For the metaphyses of the 3‐month‐old animals, the trabecular region of interest (ROI) extended from 40 μm to 936 μm away from the growth plate, along the long axis of the bone. For the older animals, the length of this trabecular ROI was scaled by the ratio of the average bone length for the age group to the average bone length for the 3‐month‐olds so that the ROI size would remain anatomically proportional as the animals grew. For the vertebral body, the trabecular ROI extended from 40 μm cranial to the caudal growth plate to 40 μm caudal to the cranial growth plate. For the diaphyses, the cortical ROI was an 800‐μm‐long segment that was centered at the mid‐diaphysis.

All animal experiments were approved by the Institutional Animal Care and Use Committee at Boston University. Micro‐computed tomography (microCT), transcriptomic, histological, and cell culture studies comparing male to female aging were all performed in the same mice (*n* = 26 males, *n* = 36 females), which were obtained from a C57BL/6 colony maintained at Boston University and housed under a 12‐hour light/dark cycle. All analyses on these mice were performed at 3, 6, 9, 12, and 18 months of age. A second set of 3‐month‐old female C57BL/6 mice (*N* = 6) was obtained from Jackson Laboratory (Bar Harbor, ME, USA) and was used as an external control for this study. A schematic of the work flow of the experiments is presented in Fig. 1 and summarizes the set of experiments performed with this core set of 68 mice. The schematic further outlines the elements of the major phenomic and transcriptomic analysis that are outlined in the experimental methods below. The experiment presented in Fig. 7 was from a third group of 8‐week‐old male and female (*N* = 4) C57BL/6 mice obtained from Jackson Laboratory.

**Fig 2:**
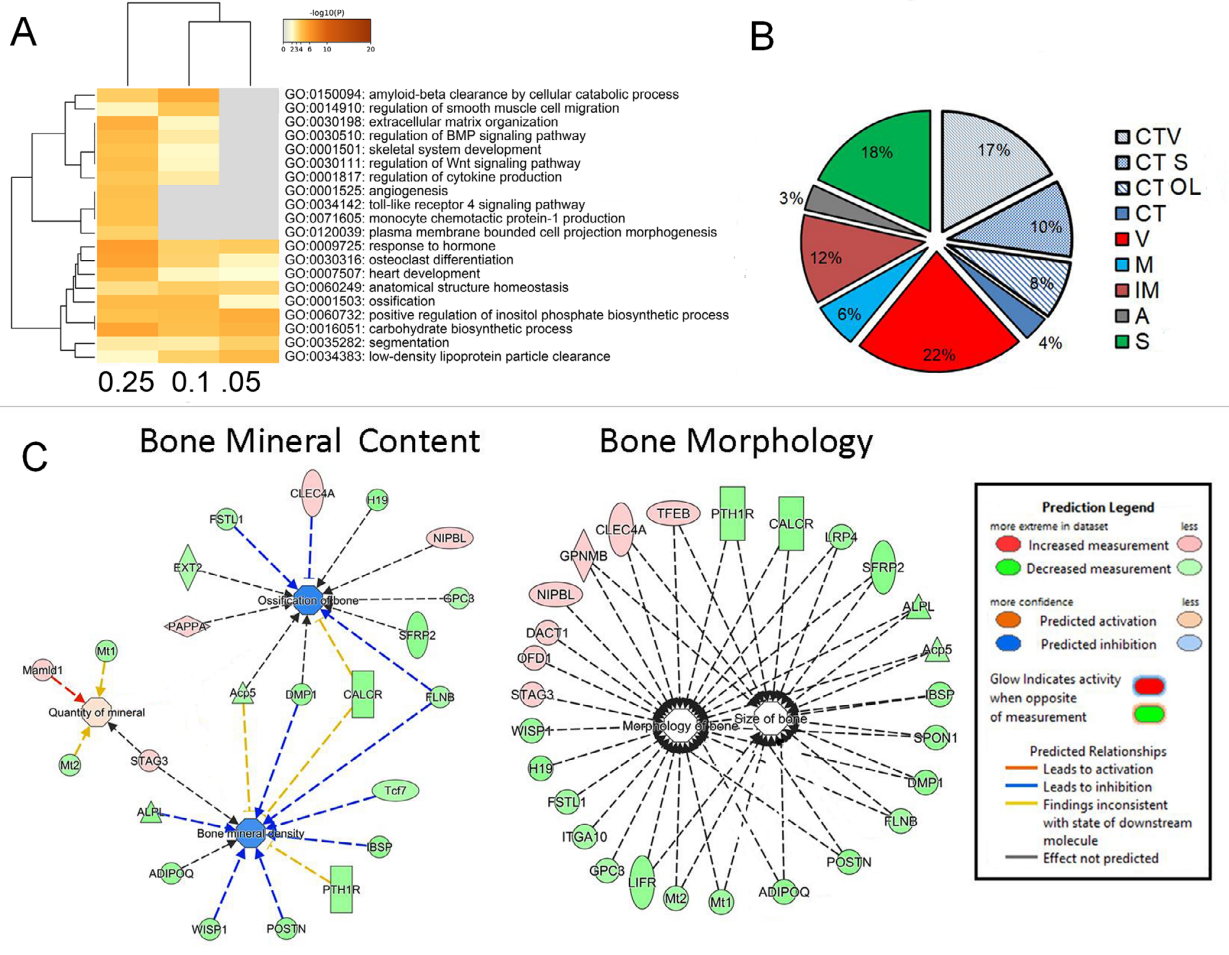
Characterization of the sexually dimorphic genes that show differential expression in bone tissue and their association with specific biological and environmental perturbations that have both skeletal and non‐skeletal effects. (*A*) Comparative ontology assessment across different statistical stringencies of the differential gene expression. Heat maps are from the enrichment analysis carried out in Metascape for DE having high FDRq 0.05, FDRq 0.1 to low FDRq up to 0.25. The heat map cells are colored by their *p* values as shown in the figure. (*B*) Percentage tissue distribution is based on ontological association with six tissues: CT = connective tissues; S = skeletal; A = adipose; IM = immunological; M = muscle; and V = vascular. Ontological association to connective tissues is denoted with light blue patterned pie sections and is separately labeled if the genes were also associated with skeletal tissues, vascular tissues, or both tissues CT OL (overlap). A group of connective tissue genes not assigned to any one tissue type is denoted CT (dark blue). A very small number of CT‐associated genes were also associated with A, IM, and/or M and are not represented in the pie chart. (*C*) Network interactions within two primary functional gene groupings within skeletal tissues were constructed using IPA. These groupings were determined from merging 10 gene sets in the total 353 genes. Gene sets from diseases and functions are white nodes, and functional pathways are denoted as blue nodes. Legend for these groupings, predicted network interactions between the genes and molecular functions for gene symbols are indicated. Color coding for the gene expression are based on ratio of the mean male values to the mean female values.

### mRNA isolation and RT‐PCR analysis

RNA used for all analysis was from the pooled humeri from each mouse and came from a larger study comparing male and female mice in wild‐type and heterozygote mice carrying one function allele of the BrD2 gene. Quality control assessments for the RNAs used in this study are completely summarized in Supplemental Table S1 and the Supplementary Appendix S1 of this article. Joint surfaces were removed from each bone and total RNA was processed from mid‐diaphysis tissues inclusive of marrow and periosteum. Total RNA was prepared from bones and cell cultures, and qRT‐PCR was carried out as previously described.^(^ ^19^, ^20^ ^)^ Each qRT‐PCR reaction was performed in duplicate or triplicate for 4 to 8 animals per age/sex group, except for studies depicted in Fig. 7*A*, in which only one sample was available for 9‐ and 12‐month‐old male and female groups. qRT‐PCR reactions were performed and analyzed using a ABI 7700 Sequence Detection System (Applied Biosystems, Foster City, CA, USA). The C_T_ values of 18S rRNA were used for normalization.

### Microarray analysis

All microarray laboratory procedures were performed at the Boston University Microarray and Sequencing Resource. Raw CEL files and RMA normalization were as previously described.^(^ ^21^, ^22^ ^)^ Raw cell files and RMA‐normalized gene expression values have been deposited in the Gene Expression Omnibus (GSE141451). Methods for sample processing, normalization of microarray data, and quality control measures for examining for batch effects and comparing individual samples by principal components are fully described in the Supplementary Appendix S1.

#### Identification of sex‐specific signature of autosomal gene expression

The effect of sex on gene expression was determined by performing Student's two‐sample *t* test (assuming equal variance) across all time points in a subset of 37 wild‐type male and female animals, using non‐log‐transformed expression data (Supplementary Appendix S1). The group of genes found to be differentially expressed (as defined by a false discovery rate [FDR] *q* < 0.25) between sexes was used for initial ontology assessments and is summarized in Supplemental Table S2. A unique, pattern‐based search program (openSESAME [Search of Expression Signatures Across Many Experiments])^(^ ^23^ ^)^ was used to identify data sets within the NCBI Gene Expression Omnibus (GEO) that display coordinate differential gene expression of the sex‐specific expression gene set that was identified in bone. Full details of this approach are found in the Supplementary Appendix S1, as well as a summary of the rodent data sets identified by this analysis (Supplementa1 Table S3).

#### Identification of sex specificity in temporal gene expression patterns

Temporal clustering of gene expression was performed using an entropy penalized expectation maximization (EPEM) algorithm with an underlying mixed‐effects model using a third‐order fixed‐effect (FE) polynomial and a second‐order gene‐specific random‐effect (RE) polynomial to allow for gene‐specific heterogeneity within a cluster.^(^ ^24^ ^)^ Data were averaged over the four replicates and standardized by the gene‐specific mean and standard deviation at each time point. The analysis was then performed on these data, treating temporal gene‐expression profiles from males and females as distinct objects that were then analyzed jointly in a single EPEM. Although a common approach is to cluster the data for each sex separately and compare the two sets of clusters, using EPEM, different cluster numbers may be obtained for each sex, and cluster labels need to be remapped as labels, which are then assigned independently. Therefore, our approach clusters the data together such that temporal gene expression profiles across sex are treated as distinct objects, allowing us to determine if genes across sexes are partitioned into the same or different clusters. The entire data set generated from this analysis is presented in Supplemental Table S4.

#### Ontology assessment and pathway analysis

Ingenuity pathway analysis (IPA) software (http://www.ingenuity.com/) and Metascape^(^ ^25^ ^)^ (https://metascape.org/gp/index.html#/main/step1) were used for all biological and functional assessments of gene groups identified by the various computational approaches used in this study. Only ontology groupings with modified Fisher exact test *p* < 0.05 were considered to be significantly overrepresented for these assessments. For the analysis using the *t* test approach, the entire group of genes in Supplemental Table S2 was assessed. For the analysis using the EPEM algorithm, individual gene groups were manually collated, and only genes associated with overrepresented ontologies related to skeletal tissues, skeletal cell types, skeletal development, skeletal morphology, and skeletal pathologies were assessed (Supplemental Table S5B).

### Micro‐computed tomography (microCT)

At euthanasia, bones (*n* = 4–10 right tibias per age and sex; *n* = 4–11 L_5_ vertebrae per age and sex) that were used for microCT were immediately placed in 4% paraformaldehyde for 1 week at 4°C, after which the bones were stored in phosphate‐buffered saline at 4°C. Samples were scanned at a nominal resolution of 8 μm/voxel and 16 μm/voxel for analysis of trabecular and cortical bone, respectively. Standard trabecular and cortical parameters then were calculated.^(^ ^26^ ^)^ Full details of the microCT analysis are fully described in the Supplementary Appendix S1 and Table S6.

### Histology

After completion of the micro‐CT scans, a subset (*n* = 5) of tibias per time point and sex was selected for histological analysis. Bones were processed, blocks were sectioned, and histomorphometric analysis was carried out as previously described.^(^ ^27^ ^)^ Histomorphometry measurements for osteoclasts were made from 5 slides per mouse from the same bones as used for microCT. Periosteal measurements were made from 8‐week‐old male and female mice (*n* = 2 per sex). The analysis of periosteal tissues was performed of femurs from 8‐week‐old C57BL/6 mice (*n* = 2 per sex). Tissues were processed in the same manner for standard histomorphometry. Details of these analyses and immunological analysis for cathepsin K antibody staining are fully described in the Supplementary Appendix S1.

### Cell culture

Bone marrow stromal cell populations were prepared from individual mice in the Boston University colony at 3, 6, 9, and 12 months of age. Cells were collected from both femora and the tibia contralateral to those used for the microCT and histological assessments.^(^ ^20^ ^)^ In vitro osteogenesis cell analysis represents independent bone marrow preparation and measurements from 3 cell preparations per time point per age. Differentiation was performed as previously described.^(^ ^20^ ^)^ Data were grouped by two age groups (3–6 months and 9–12 months) for analyses.

### Statistical analysis of phenomic data

Using multiple linear regression, the effects of bone site, age, sex, and their interactions were assessed for each of the microCT outcome measures (SAS 9.3, SAS Institute Inc., Cary, NC, USA). For assessments of cell culture studies, statistical analyses were performed with Prism 5 (GraphPad Software Inc., La Jolla, CA, USA), and data are presented as mean ± standard error (SE). Gene expression data from the in vitro experiments were log transformed and then compared using two‐factor ANOVA (Bonferroni, with sex and medium as factors). The sample sizes for each analysis are indicated in the figure legends.

## Results

### Sex‐associated quantitative transcriptomic differences in skeletal tissues

#### Sex‐specific differential gene expression

Using a liberal FDR *q* cut‐off (FDR *q* < 0.25, *p* < 0.005), 353 differentially expressed genes (DE) genes between males and females were identified (Supplemental Table S2). Two separate omics analysis programs, Metascape and IPA, were used as a means to independently assess these genes and confirm their associated biological, physiological, and cellular functions. We first compared the DE gene groups with different statistical stringencies of selection (FDR 0.05, 0.1, and 0.25 groups) using the Metascape program to validate that even as stringency of selection was loosened, the same overlapping biological functionalities were observed between the groups. Thus all three groups showed the same nine ontologies inclusive of genes associated with ossification osteoclast function and tissue morphogenesis (Fig. 2*A*). The ontology gene association with hormone function was related to insulin activity (GO 0009725), whereas within the overlapping groups at the lower stringencies, genes associated with bone morphogenetic protein (BMP) and Wnt signaling were identified. Finally, it is interesting to note the most stringent FDRq (0.05) group contained PTHr, 1 CalcR, and Sfrp2, suggesting that there are both sex‐linked differences in cellular responses to systemic mineral metabolism and morphogenetic responses to Wnt signaling.

**Fig 3:**
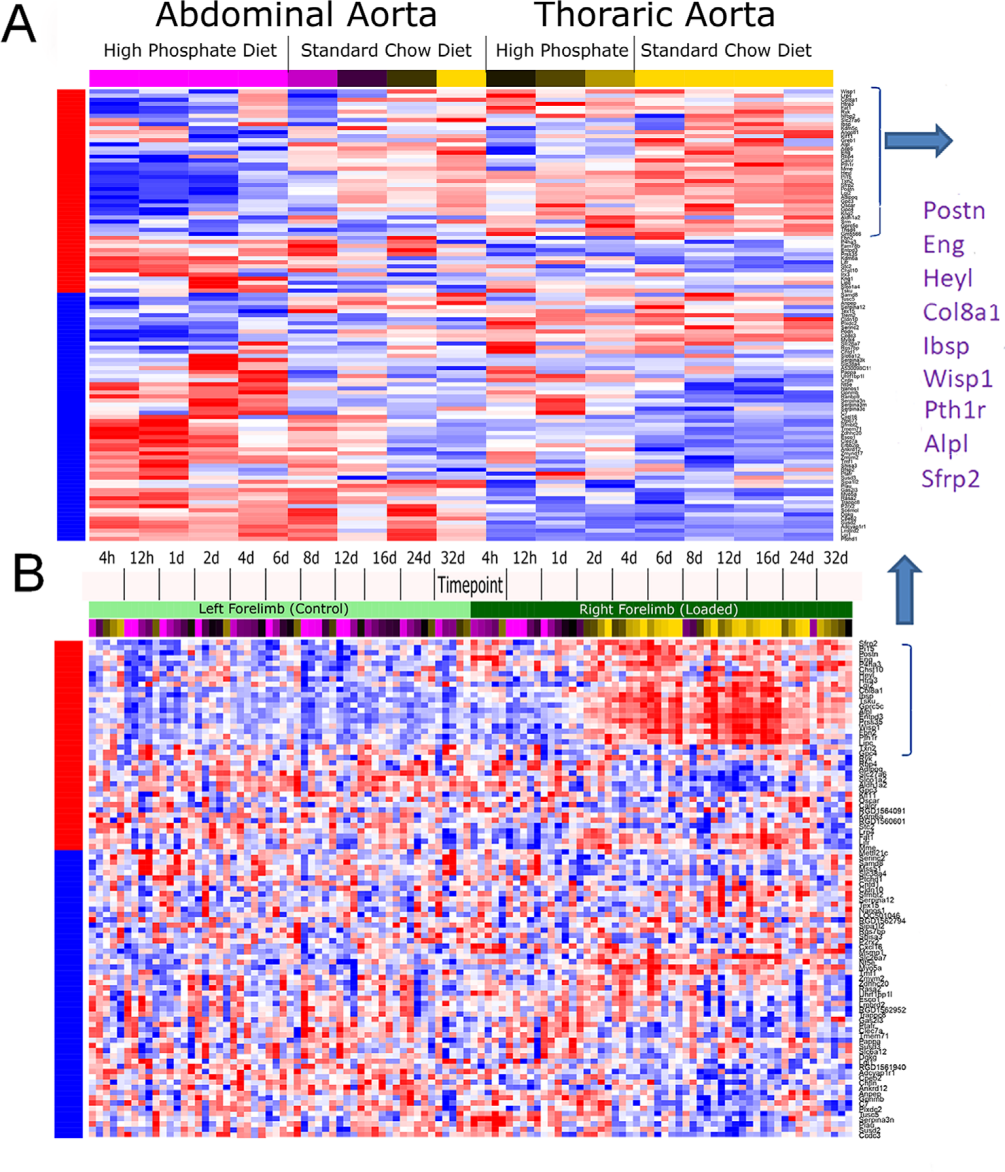
OpenSESAME analysis displaying two of 28 rodent gene sets that showed association to the coordinate sex‐specific gene signature of bone. (*A*) The coordinate sex‐specific gene signature association with the gene expression changes found in aortic arterial calcification. (*B*) The coordinate sex‐specific gene signature association with the gene expression changes found in in vivo forelimb mechanical loading. Experimental conditions are shown across the top of the two heat maps. Red denotes increased and blue decreased expression from the mean chip value for each experiment. The red and blue side bars indicate the expression ratio values relative to the male versus female mice. The genes showing the greatest difference in expression as seen in the heat maps in each of the experimental series and that overlapped between the two series are bracketed in the figure. The specific genes that overlap between both of the series are listed in purple text.

We next used the IPA program as an independent means to validate gene association of the DE genes and provide a more granular assessment of specific ontological associations associated with skeletal tissue growth and mineral metabolism. These genes were primarily associated with six tissues (Fig. 2*B*): skeletal, vascular, muscle, adipose, immunological, and connective tissue. Thirty‐nine percent were associated with connective tissue, and of these, 90% were also associated with skeletal tissue, vascular tissue, or both. In contrast, very few of the genes that were associated with connective tissue were also associated with muscle, adipose, or immunological tissues. Interestingly, vascular and skeletal tissues had nearly equal representation, involving 22% and 18% of the genes, respectively.

Consistent with the gene ontologies identified in Metascape, numerous genes that were related to skeletal tissues were associated with ontologies that could be described as falling into two broad categories: those related to bone morphology and size and others related to bone mineral content. Fig. 2 shows a selective set of these ontologies that are diagrammatically presented to show the relative expression of the male‐to‐female ratios of the genes identified in these ontologies. Other ontologies that are not shown are primarily related to the cellular activities of bone (remodeling and bone cell regulation). The primary biological ontologies and upstream regulators and targets that were associated with the DE genes are presented in Table 1. The most prevalent physiological systems, developmental processes, and functions were consistent with the tissue distributions presented in Fig. 2. The upstream regulators with the greatest significance are all implicated with aspects of skeletal tissue development or skeletal tissue aging. For example, miR‐218‐3p is noteworthy given that it regulates multiple aspects of Wnt activity through its actions on several primary targets known to regulate bone cell activities, and because multiple Wnt ligands and receptor genes associated with stem cells have elevated and earlier temporal expression in females (see below). Another example, Src has been shown to be a central transcriptional regulator of osteoclast development and functions as well as a key upstream regulator of multiple osteoclast targets.^(^ ^28^ ^)^

**Table 1:** Quantitative Sex Associations of Biological System and Molecular Functions

Physiological system development and function		
Ontological grouping	*p* value^a^	No. of genes^b^
Connective tissue development and function	1.72E‐02–1.71E‐06	57
Tissue morphology	1.74E‐02–1.71E‐06	91
Skeletal and muscular development and function	1.58E‐02–6.08E‐06	62
Tissue development	1.74E‐02–6.08E‐06	98
Cardiovascular system development and function	1.74E‐02–6.16E‐06	57

Upstream regulator	*p* value of overlap^c^	Primary targets^d^
Mir218 3	7.6E‐05	SFRP2, SOST, Tcf7
ZNF106	6.78E‐04	ACTC1, GLRX, GPNMB, PMP22, ERPINA3
SRC	9.79E‐04	Acp5,DC‐STAMP
KDM8	7.32E‐04	MYC, POSTN, PTH1R
TP73	1.00E‐03	ATG4C, CLEC7A, CYR61, DMC1, FAM78
		GPNMB, SERPINA,TREM2

^a^ The lowest and highest *p* values (for modified Fisher exact score) for the genes associated with the ontological grouping. The *p* values <0.05 indicate significant representation of the ontological grouping in the list of 353 genes that showed different expression in males than females over all time points. The range is based on *p* values for genes in the group in the whole genome versus the number of genes in group size that was screened.

^b^ Number of genes of the 353 assayed associated with the ontological grouping.

^c^ The *p* values for modified Fisher exact score indicating ontology group membership based on the number of molecules in the group that would be regulated by the predicted upstream regulator.

^d^ Primary targets of the upstream regulator identified target molecules in the ontology group.

#### Sex‐specific differential gene expression is systemic and associated with multiple biological processes

We next assessed whether bone‐specific sex DE genes showed a more systemic prevalence in their sex‐specific expression in other tissues and were potentially associated with other biological mechanisms. These questions were addressed using a pattern‐based approach, openSESAME, to search the publicly available data sets of the GEO repository for other experimental and/or biological conditions that show regulatory changes in this same set of genes. This approach assigns a signature association (SA) score to each sample in each data set, with positive and negative SA scores indicating whether the genes in the signature are coordinately regulated with respect to the other samples in the data set in the same or opposite manner, respectively, as in the original signature. Each data set is then assessed using Fisher's exact test to determine whether it is significantly enriched in samples with strong positive and negative SA scores. For this analysis, we used the DE group having FDR *q* < 0.1. This set of genes encompassed 51 up and 60 down genes that were significantly regulated in male mice relative to female mice. A search of all GEO repository data sets identified 28 rodent series that showed significant coordinate expression of the sex‐specific bone signature. A summary of what these 28 data sets are and how we carried out these studies is more extensively summarized in the Supplementary Appendix S1 (Table S3).

Two data sets from the 28 were selected for further analysis because they addressed research questions regarding connective tissue formation and/or mineral regulation of bone tissues in female mice. In the first data set (GEO Series GSE57818), gene expression was profiled in the aortic and thoracic arteries of female DBA/2 mice maintained on a standard or high‐phosphate diet for 14 days.^(^ ^29^ ^)^ This data set is significantly enriched in samples with strong positive or negative association with the sex‐specific gene expression signature (Fisher *p* = 0.0007, FDR *q* = 0.128) (Fig. 3*A*). A subset of the genes in the signature, which we had found to have higher expression in female bones, was much more strongly downregulated with high‐phosphate diet in the abdominal aorta (which undergoes calcification in response to the high‐phosphate diet) than in thoracic aorta (which does not). In the second study (GEO Series GSE22286), the right forelimb of female rats was loaded axially for 3 minutes per day, and gene expression was profiled in both forelimbs over a time course of 4 hours to 32 days.^(^ ^30^ ^)^ This data set was significantly enriched in samples with strong SA scores (Fisher *p* = 7.24e−07, FDR *q* = 0.00037) (Fig. 3*B*), driven largely by a subset of genes that were strongly upregulated over time in the loaded limb compared with the contralateral control. Interestingly, a small subgroup of genes, which we had found to have lower expression in male bones, was strongly regulated in both experiments (purple text in Fig. 3*A*, right). These genes predominantly encode connective tissue matrix proteins (*Postn*, *Ibsp*, *Col8a1*, *Wisp1*) and have been specifically associated with regulation of mineral metabolism (*Pthr1*) and Wnt regulation (*Sfrp2*). Four of these genes had an overall sex‐specific difference in their expression with FDRq <0.05 and *p* values <0.00018 and two with FDRq <0.07 and *p* values <0.0003 (Supplemental Table S2).

**Fig 4:**
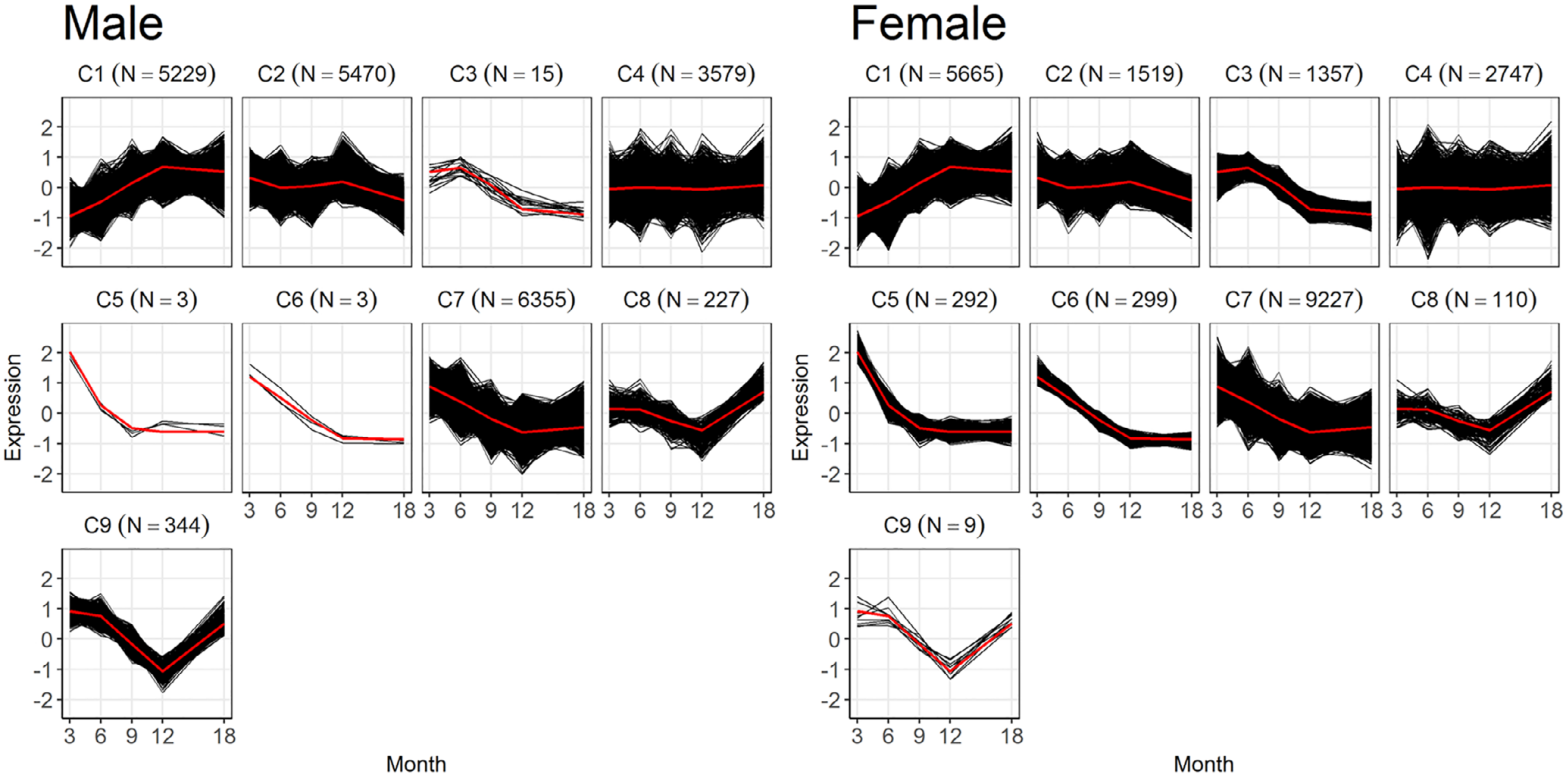
Temporal clustering of male and female bone transcriptomes overall and in relation to selective gene ontologies associated with skeletal morphology, coupled remodeling, estrogen metabolism, and reproductive capacity. Cluster plots obtained by the entropy penalized EM clustering algorithm of gene‐expression profiles for male and female expression profiles. Cluster numbers are denoted C1, 2, etc., while the number of genes contained in the cluster is indicated in parentheses. Individual panels show the spread of expression data for all genes (black) in the cluster as well as the mean expression (red) of all genes in the cluster. The gene sets for the cluster data from the EPEM analysis and the cross‐tabulations of the cluster labels in male and female mice are in Supplemental Table S4.

### Sex‐specific temporal differences in transcriptome during postnatal bone homeostasis and aging

#### Clustering results

We next assessed the temporal difference in gene expression between male and female mice using a newly developed EPEM clustering algorithm.^(^ ^24^ ^)^ Nine predicted clusters with unique temporal patterns of gene expression were obtained (Fig. 4). Cross‐tabulations of the cluster labels in male and female mice were obtained to determine genes that showed concordance (common) or discordance (discordant) in temporal patterns of gene expression levels between the two sexes. The gray on‐diagonal entries of Table 2 correspond to genes that were grouped into the same cluster in both sexes (common temporal expression). As examples, the largest cluster, clusters 1, 4, and 7, had 4353, 1009, and 5023 genes, respectively, that showed the same temporal patterns of expression between the male and female animals. The off‐diagonal entries correspond to genes that showed different patterns and were then assigned to different clusters in male versus female mice and thus are genes showing temporal discordance in expression between the two sexes. For example, 1166 genes for female mice showed on average an increase in expression levels until 12 months of age followed by relatively constant expression (cluster 1), whereas in the males, the expression levels for these genes were largely unchanged over time (cluster 4).

**Fig 5:**
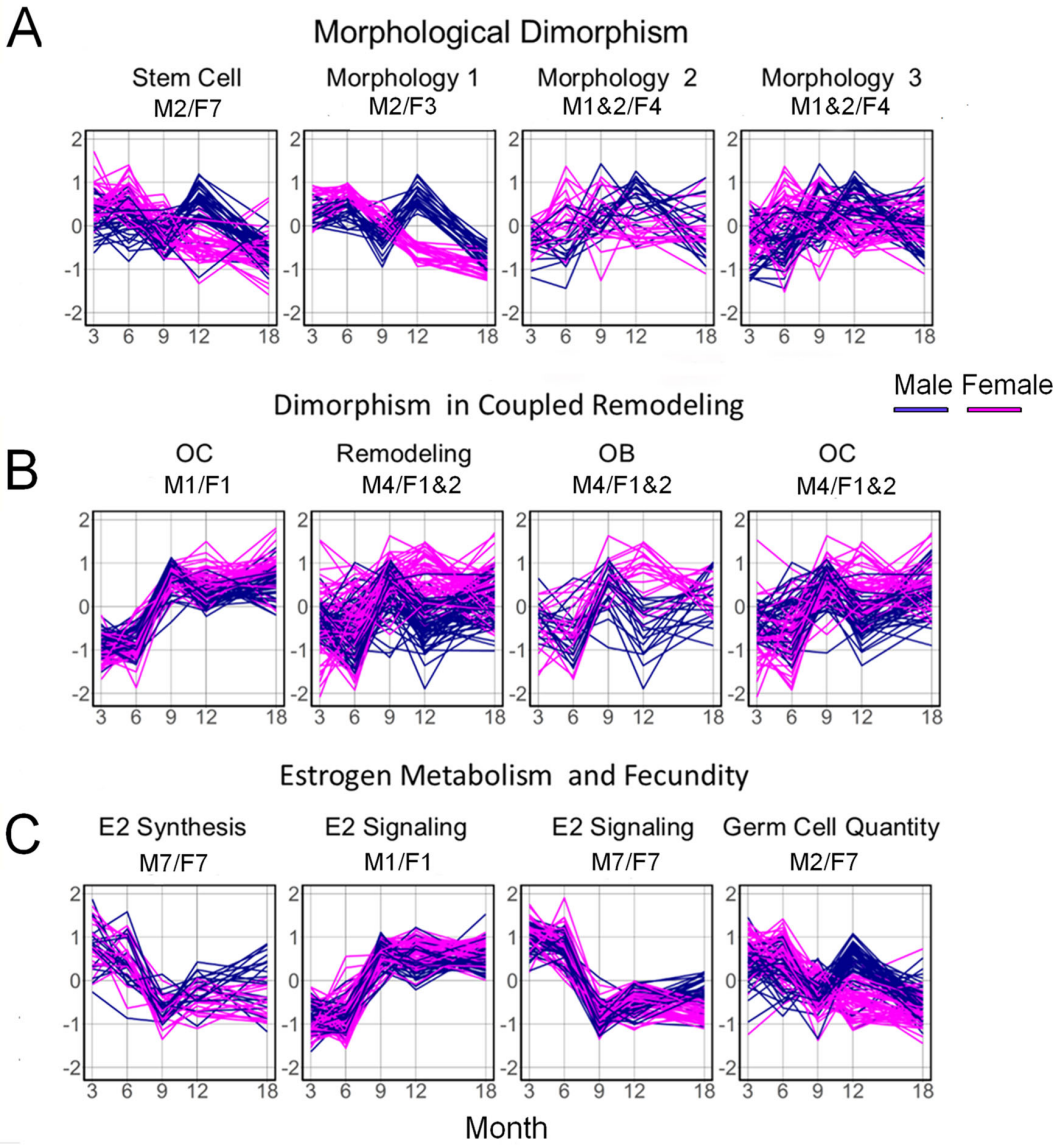
Selective temporal gene sets identified as having significant overexpression to skeletal tissue functional ontologies (morphology, remodeling/osteoblast of osteoclast function), estrogen biosynthesis, estrogen signaling, and oocyte production. Ontology groups were manually curated based on their descriptors in IPA. Expression patterns of the selected sets of genes that were associated with (*A*) morphological dimorphism, (*B*) coupled remodeling and bone cell activity, and (*C*) estrogen metabolism and fecundity are presented. The same sets of genes in male (blue) versus female (red) mice are plotted over time. The male and female gene sets that were derived are denoted in the figure and may be cross‐referenced to the data in Supplemental Table S5. A full listing of these genes and the ontologies used to organize them into sets are provided in Supplemental Table S5B.

**Table 2:** Common and Discordant Temporal Profiles of Gene Expression in Males and Females

		Female gene‐expression curves								
Male gene‐expression curves		1	2	3	4	5	6	7	8	9
1		4353	278	10	504	0	0	80	4	0
2		80	530	1064	471	126	141	3032	26	0
3		0	3	0	1	0	0	11	0	0
4		1166	396	42	1009	54	13	860	35	4
5		0	0	0	0	0	0	3	0	0
6		0	0	0	0	0	0	3	0	0
7		16	263	239	519	112	144	5023	34	5
8		41	25	0	111	0	0	46	4	0
9		9	24	2	132	0	1	169	7	0

Distribution of the 21,225 genes assigned to the same (on‐diagonal, common) and different (off‐diagonal, discordant) clusters. Clusters were obtained from a single run of the entropy penalized EM clustering algorithm including all genes from both male and female mice to obtain nine clusters. In Supplemental Table S3, each gene is listed with its assigned female and male cluster number(s).

#### Ontology and pathway assessment

Based on those genes that shifted between various temporal expression patterns, the overall transcriptome was divided into two nearly equally sized subsets: 10,919 genes were in groups on the gray diagonal (common temporal profiles) and 10,306 genes were in groups on the off‐diagonal (discordant temporal profiles). The genes in each group with gene ontologies that both (i) are related to skeletal tissues, skeletal cell types, skeletal development, skeletal morphology, or skeletal pathologies; and (ii) have significant overrepresentation (modified Fisher exact *p* < 0.05) in the entire data set were then selected. A total of 705 genes were selected out of the total transcriptome: 269 of these genes (38%) were in common groups compared with 436 (62%) in the discordant groups (Supplemental Table S4). Hence, while the total transcriptome was almost equally divided between genes with common versus discordant temporal profiles in males and females, a larger proportion of the skeletally associated genes show discordant temporal profiles. In the context of using this study as a reference, the data in Supplemental Table S4 may be accessed by interested readers for their own ontology assessments. This may be done by identifying gene groups that show discordance in the cross tabulation table then relating how that discordance is related to shift in the pattern of gene expression found by relating these differences to graphical profiles in Fig. 4. The tabulation of the various genes that have shifted their pattern of expression may then be identified in Supplemental Table S4. As an example, two differences that we noted relate to both differential and temporal variations between males and females in insulin and fat metabolism and inflammation and immune cell functions (data not shown). These data then may be used to identify candidate genes associated with inflammation and both innate and adaptive immune functions, which are also involved with skeletal pathology.

As with the genes that showed the most significant differential expression between males and females irrespective of time point (Fig. 2; Supplemental Table S2), those that showed temporal differences in expression between males and females were associated with connective tissues and development of both skeletal and vascular tissues (Table 3). Aspects of numerous ontology pathways associated with various skeletal cell types and pathologies, innate and adaptive immunity, and inflammation were associated with the temporal differences in expression between males and females. Interestingly, while dendritic cell–associated activities were found only in the common temporal group, T‐helper cell (Th1 and Th2)‐associated activities that are associated with the development of autoimmunity^(^ ^31^ ^)^ were discordantly expressed between the sexes. The primary upstream regulator associated with osteoclast differentiation *Tnfsf11* (RANKL) was also in the common group, while both *Tnf*, a major driver of numerous skeletal inflammatory diseases, and *Ctnnb1*, a primary intracellular signal transducer of Wnt signaling, were in the discordant group **(**Table 3).

**Table 3:** Common Versus Discordant Temporal Expression Patterns Between Males and Females for Physiological System and Development Functions, Canonical Pathways, and Upstream Regulators

Physiological system development and function		
Name	*p* value (upper ranges)^a^	No. of molecules common/discordant^b^
Connective tissue development and function	1.07E‐17–1.21E‐162	224/375
Skeletal and muscular development and function	1.07E‐17–1.21E‐162	223/378
Tissue development	1.074E‐17–1.21E‐162	244/399
Organism survival	1.15E‐21–9.19E‐96	195/281
Tissue morphology	4.31E‐18–1.16‐91	219/0
Organismal development	9.16E‐16–1.43E‐104	0/374

Canonical systems		
Name	*p* value	Overlap^c^
Common/discordant		
Role of osteoblasts, osteoclasts, and	1.15E‐38–6.5E‐20	(36/227)/(45/227)
chondrocytes in rheumatoid arthritis		
Osteoarthritis pathway	4.18E‐29–1.55E‐20	(36/204)/(35/204)
Role of macrophages, fibroblasts, and endothelial cells in rheumatoid arthritis	1.22E‐24–1 .26E‐18	(38/312)/(40/312)
Neuroinflammation signaling	8.90E‐22–8.30E‐16	(34/285)‐(34/285)
Common only		
Dendritic cell maturation	2.65E‐24	17.5 (30/171)
Discordant only		
Th1 and Th2 activation	1.53E‐13	14.9 (25/168)

Upstream regulator	*p* value of overlap^d^	Predicted activation^e^
Common/discordant		
IFNG	4.98E‐23–1.06E‐18	–/Activated
Common only		
IKBKB	1.71E‐20	(−)
TNFSF11	6.41E‐20	(−)
APOE	1.13E‐16	(−)
Discordant only		
TNF	9.38E‐18	Activated
CTNNB1	1.75E‐17	(−)
KAT6A	5.01E‐17	Activated
RBPJ	4.99E‐14	(−)
TP53	1.13E‐18	Activated

^a^ The *p* values for modified Fisher exact score as to group membership based on associated molecule count in the groups. The range presented consists of the lowest and highest *p* values found in the common or discordant groups or individually in a single group.

^b^ Number of molecules with common/discordant temporal expression between males and females.

^c^ “Overlap” is the number molecules observed in the group per total number of molecules that are known to be associated with the pathway.

^d^ The *p* values modified Fisher exact score as to group membership based on associated molecule count in the group that would be regulated by the predicted upstream regulator.

^e^ The predicted role to the regulator is based on average value across all of the male/female expression ratios for the molecules regulated by the upstream target.

We next identified significantly overrepresented skeletal gene ontologies among the genes that were differentially expressed both quantitatively and temporally between the sexes. This analysis was performed with both the aforementioned lists of time‐independent sex‐specific differentially expressed 353 genes (Fig. 2; Supplemental Table S2) and 705 skeletal‐associated genes that are temporally expressed in a sex‐specific manner. (Supplemental Table S4). These results are summarized in Table 4. A number of these genes overlapped with the multiple pathways shown in Fig. 2*B*. Interestingly, this group included four central regulators of mineral metabolism and osteoblast and osteoclast function (*Pth1r*, *Calcr*, and *Lrp4*), as well as most of the subgroup of genes that had been identified as overlapping between the two experiments showing coordinate regulation of the sex‐specific differentially expressed genes (*Postn*, *Ibsp*, *Pth1r*, *Sfrp2*, and *Alpl*; Fig. 3*A*, *B*).

**Table 4:** Sex‐Specificity in the Transcriptome, Restricted to Gene Ontologies Associated With Skeletal Function

Common					
Accession number	Molecule name	Description	Male/female^a^	*p* value	FQR‐value
14955	H19	H19 fetal liver mRNA	0.375	1.414E‐06	0.0015
15891	IBSP	Integrin binding sialoprotein	0.421	9.191E‐05	0.03369
16880	LIFR	Leukemia inhibitory factor receptor	1.03	8.419E‐07	0.0011
18792	PLAU	Plasminogen activator, urokinase	1.99	0.0006154	0.0993
20319	SFRP2	Secreted frizzled‐related protein 2	0.826	1.316E‐06	0.00154
50706	POSTN	Periostin, osteoblast specific factor	0.427	0.000280	0.0675
59036	DACT1	Dapper homolog 1, antagonist of beta‐catenin (xenopus)	2.14	0.000713	0.1049
66102	CXCL16	Chemokine (C‐X‐C motif) ligand 16	2.36	8.166E‐05	0.0312
75766	DC Stamp	Transmembrane 7 superfamily member 4	0.760	0.001106	0.12890
83433	TREM2	Triggering receptor expressed on myeloid cells 2	3.46	1.125E‐09	6.60E‐06
232790	OSCAR	Osteoclast associated receptor	0.437	0.0001604	0.04786
13038	CTSK	Cathepsin K	0.464	0.001750	0.1588
21923	TNC	Tenascin C	0.490	0.00450	0.24010
16367	IRS1	Insulin receptor substrate 1	0.468	0.001560	0.15344

Discordant					
Accession number	Molecule name	Description	Male/female^a^		
11433	Acp5	Acid phosphatase 5, tartrate resistant	0.442	0.0001917	0.0544
11450	ADIPOQ	Adiponectin, C1Q, and collagen domain containing	0.0471	8.326E‐05	0.0311
11647	ALPL	Alkaline phosphatase, liver/bone/kidney	0.383	2.794E‐05	0.0149
12311	CALCR	Calcitonin receptor	0.404	2.759E‐05	0.015176
14314	FSTL1	Follistatin‐like 1	0.511	0.0013914	0.1423
19204	PTAFR	Platelet‐activating factor receptor	2.23	6.866E‐05	0.0294
19228	PTH1R	Parathyroid hormone 1 receptor	0.418	0.000138	0.0451
21841	TIA1	Cytotoxic granule‐associated RNA binding protein 1	1.67	0.000700	0.106191
56644	CLEC7A	C‐type lectin domain family 7, member a2	1.51	8.448E‐08	0.000165
93695	GPNMB	Glycoprotein (transmembrane) nmb	2.16	0.0002115	0.0572
213119	ITGA10	Integrin, alpha 10	0.479	0.0011509	0.132
228357	LRP4	Low‐density lipoprotein receptor‐related protein 4	0.460	0.000459	0.0850
12263	C2	Complement component 2 (within H‐2S)	1.98	0.00123	0.138
17386	MMP13	Matrix metallopeptidase 13	0.497	0.0028	0.200
14119	FBN2	Fibrillin 2	0.456	0.00048	0.087
17977	NCOA1	Nuclear receptor coactivator 1	1.706	0.0024	0.188
68427	SLC39a13	Solute carrier family 39 (metal ion transporter), member 13	0.515	0.0015	0.153
21841	Tia1	Cytotoxic granule‐associated RNA binding protein 1	1.69	0.00070	0.106
68027	TMEM178	Transmembrane protein 178	0.479	0.0013	0.1409
218772	RARB	Retinoic acid receptor, beta	1.701	0.0045	0.241
383435	MS4A7	Membrane‐spanning 4‐domains, subfamily A, member 14	1.77	0.0036	0.219
12912	CREB1	cAMP responsive element binding protein 1	1.638	0.0034	0.216
105278	CDK20	Cyclin‐dependent kinase 20	0.68	0.0050	0.250

Genes identified as ontologically associated with skeletal tissues, cell types, development, morphology, or pathology and that were also identified as differentially expressed between males and females over all time points (FDR < 0.25; Fig. 1, Supplemental Table S1). These genes are listed according to whether they showed common or discordant temporal expression in males versus females (Table 2, Fig. 2*A*).

^a^ Male/female expression ratios for the gene averaged across all time points.

In the next stage of the analysis, we investigated how specific skeletal gene ontologies that were related to molecular processes controlling bone morphology, postnatal skeletal remodeling, and estrogen functions temporally tracked over the 18 months of this analysis. The temporal patterns of expression of subsets of genes related to these processes are presented in Fig. 5*A–C* and are organized according to biological function; a full listing of these genes and the ontologies used to organize them into sets are provided in Supplemental Table S5. In many cases, genes associated with a given function showed discordant temporal expression between sexes and thus were in different clusters for males versus females. Two general patterns of temporal differences were observed. Sets of genes related to stem cell pluripotency (Fig. 5*A*) and morphological development of the skeleton showed elevated expression in young females compared with young males (Fig. 5*A*). This stem cell set contained multiple canonical Wnt ligands, Wnt receptor genes, transcriptional mediators of Wnt activity, several Bmp ligand genes, and two of the embryonic stem cell pluripotency genes found to be induced during fracture healing,^(^ ^32^ ^)^ *Pou5f* and *Nanog*. The morphology set included various members of the HOX and SOX regulatory families. The second pattern we observed was related to gene sets related to coupled remodeling and contained almost all the identified gene ontologies in the total transcriptomic data set for the known drivers of osteoclast (OC) differentiation and known genes associated with osteoblasts (OB) and osteoclast functions (Fig. 5*B*). These genes generally showed higher expression in females than males at 12 months, and in most cases, expression was elevated at 6 months as well. Although the expression of genes associated with germ cell production (Fig. 5*C*) is not directly carrying out this function in bone, we inferred that their expression is reflective of that within the germ cell‐producing tissues. The female patterns of expression followed those of estrogen synthesis, while the male patterns of expression were more constant over time. These results were independently confirmed by performing a comparative analysis of the concordant and discordant genes sets in Metascape. Consistent with the IPA findings, the discordant gene set showed a larger number of genes and association with limb morphogenesis, insulin hormone response, and wnt signaling, whereas the genes in both the common and discordant groups were equally and primarily associated with mineral metabolism and osteoclast function (Supplemental Fig. S3).

**Fig 6:**
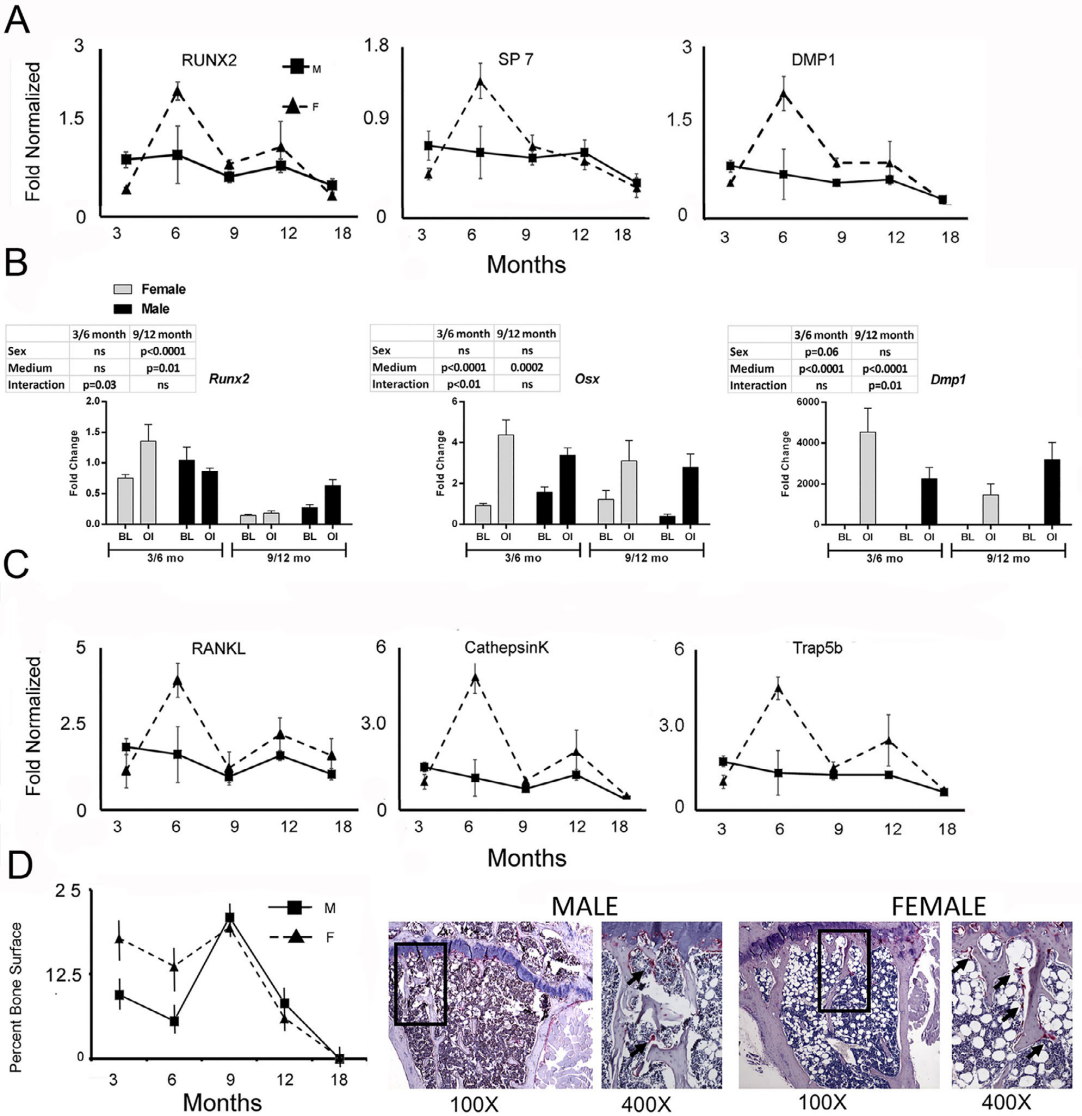
Sex‐ and age‐associated phenomic changes in osteoblast and osteoclast activities in mice. qRT‐PCR was performed on the same RNA as used for the microarray analysis. Candidate genes that were assayed are denoted in the figure. RNA analysis was made on four RNA samples from four mice from each experimental group, except for studies depicted in (*E*) in which only one sample was available for 9‐ and 12‐month‐old male and female groups. For in vitro analyses, MSCS analysis represents independent bone marrow preparation and measurements from three cell preparations per time point and age. Histomorphometry measurements for osteoclasts were made from five slides from five separate mice per experimental groups and are the same samples as used for microCT. Periosteal measurements were made from 8‐week‐old male and female mice (two per sex). (*A*) Candidate RNA analysis for osteoblast activity. Data are presented as means ± SE (*n* = 4–6). (*B*) Comparison of osteogenic potential of male and female bone marrow MSCs. Primary bone marrow cultures were established from male and female mice (3, 6, 9, and 12 months old, C57BL/6). Cultures were either harvested on day 7 at their basal condition (BL) or after they underwent osteogenic induction (OI) for 7 days. Inset tables indicate the sex, media, and sex*media interaction for significance of the mRNA expression data (two‐factor ANOVA, Bonferroni). (*C*) Candidate RNA analysis for osteoclast activity. (*D*) Male and female C57BL/6 mice are dimorphic for changes in osteoclast activity with age. Tibias were harvested and fixed from male and female mice (3, 6, 9, 12, and 18 months old, C57BL/6 male and female mice). After micro‐CT scanning, tibias were decalcified, embedded, and sectioned. Osteoclasts were identified by TRAP staining and active osteoclast surface was analyzed. Data are presented as means ± SE (*n* = 4). *p < 0.05 (*t* test between matched time points. Candidate RNA analysis. Humeri were harvested from the same mice that were analyzed by micro‐CT; the same RNA as isolated from whole bone and presented for the microarray analysis was used from these analyses.

### Phenomic sex‐ and age‐associated changes at the tissue, cellular, and organ levels

#### Resorption is higher and temporally associated with the period of sexual reproduction in females

Correspondence between the sex‐specific transcriptomic differences and the functional activities of specific skeletal cell populations in bone was next examined. Osteogenesis was examined by assessing the expression of mRNAs representative of osteogenesis and lineage progression (*Runx2, Sp7*, *and Dmp1*) within intact bones (Fig. 6*A*). These results showed that female mice had higher levels of expression of the markers for osteogenesis that peaked at 6 months; however, by 9 months, levels had fallen back to close to those of the male mice. The cellular differentiation potential of marrow stromal stem cells was assessed in vitro by growing these cells in either basal media absent differentiation inducers (BL) or in conditions that promoted osteogenic differentiation (OI) (Fig. 6*B*). At 3 and 6 months of age, *Runx2* expression was comparable between sexes in both the basal and osteoinductive media, whereas at 9 and 12 months of age, *Runx2* expression was higher in males than females. This sex‐associated difference in the cultures from older animals, particularly in the presence of osteoinductive media, suggests a greater ability of the male cells to commit to the osteogenic lineage. Both male and female cultures showed equal ability to progress to their osteoprogenitor stage based on their expression profiles of *Sp7* (Osterix); however, younger female cells expressed greater levels of the osteocyte marker *Dmp1* than male cultures when grown under osteoinductive conditions.

**Fig 7:**
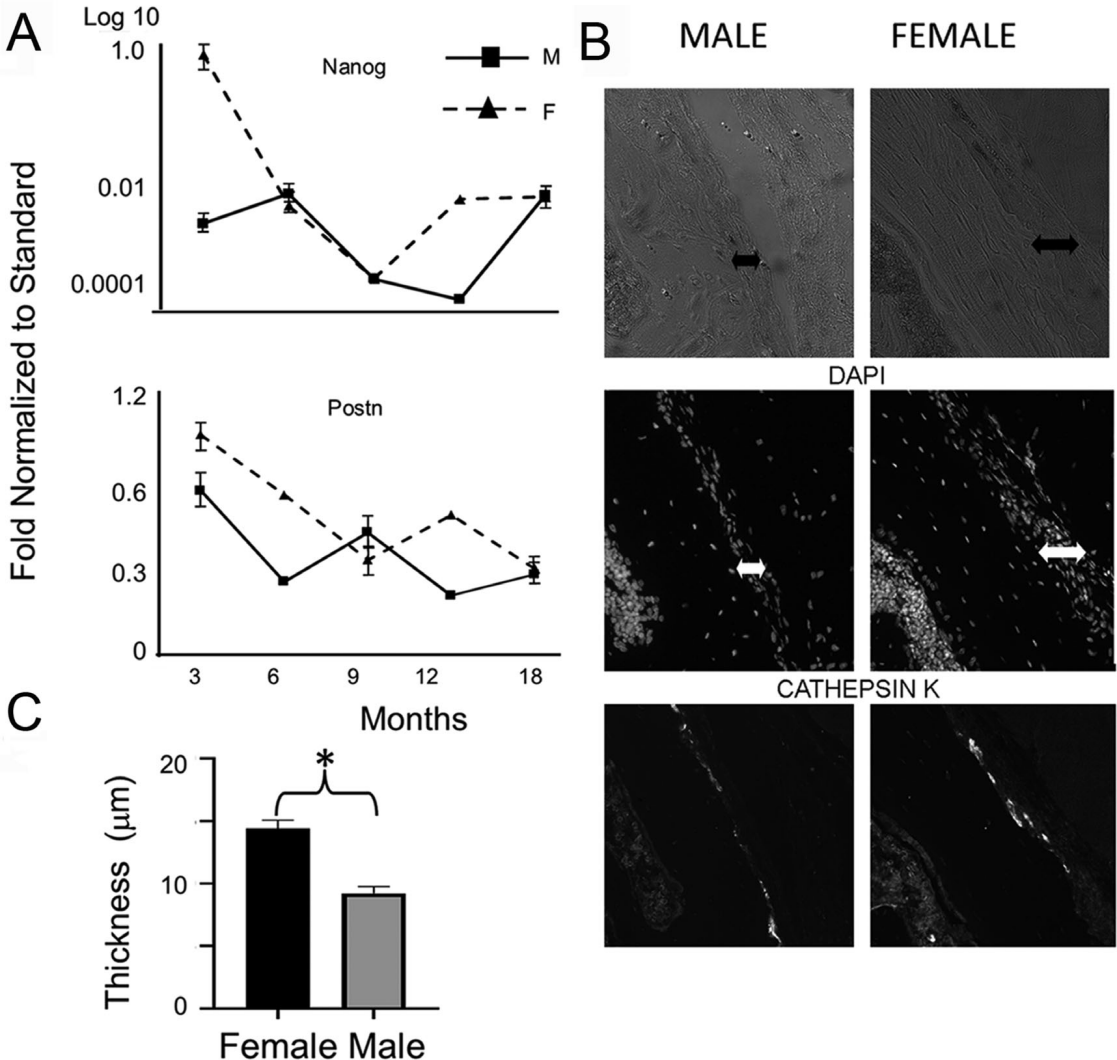
Appositional growth markers occur earlier and at higher levels in females than males. (*A*) Candidate RNA analysis for periosteal stem cells and periosteal progenitor activity. (*B*) Mean periosteal thickness of male and female mice. Unpaired *t* tests were used to compare sections from different sexes.* = (± SD, *p* < 0.01). (*C*) Representative immunohistological sections for periosteal evaluation for skeletal stem cells expressing cathepsin K. Sex of animals and nature of fluorescence image are indicated in the figure. Arrows denote observed width of the periosteum in the image.

Osteoclast activity was next examined by assessing the expression of mRNAs representative for these cells and their activity (*Tnfsf11*/RANKL*, Trap5b*, and *Ctsk*). The temporal expression profiles of these mRNAs followed those found for osteoblasts; however, these mRNAs were always expressed at higher levels in females over the entire 18 months (Fig. 6*C*). Static histomorphometry of bone surface area undergoing active resorption was used as a measure of the overall remodeling taking place in both male and female mice (Fig. 6*D*). Consistent with the mRNA data, these data also showed that the female mice had higher levels of remodeling for the first 9 months, while male mice had fairly constant but lower levels. Both male and female mice showed rapid diminishment in the surface of bone covered by osteoclasts after 12 months.

#### Appositional growth is higher and earlier in females

To assess if appositional growth on the periosteal surfaces was different in male and female mice, expression of *Nanog* and *Postn*, genes that are selectively expressed in periosteal skeletal stems cells^(^ ^33^ ^)^ and differentiated periosteal cells,^(^ ^34^ ^)^ respectively, were assessed. Female mice showed peak expression for both these mRNAs at higher levels than male at the earliest time point (3 months), whereas at later times these genes diminished in both sexes but did not show large differences between the sexes (Fig. 7*A*). Differences in female appositional growth were next examined at 8 weeks of age by determining the periosteal widths (Fig. 7*B*, *C*) and for the presence of cathepsin K‐stained cells (Fig. 7*B*) that are associated with periosteal skeletal stem cells.^(^ ^33^ ^)^ Female mice had thicker periosteum. The expression of cathepsin K in the periosteum appeared higher in the females, although the number of cells expressing this marker appeared similar between the sexes.

#### Organ‐level structural and material properties are sexually dimorphic over time

**Fig 8:**
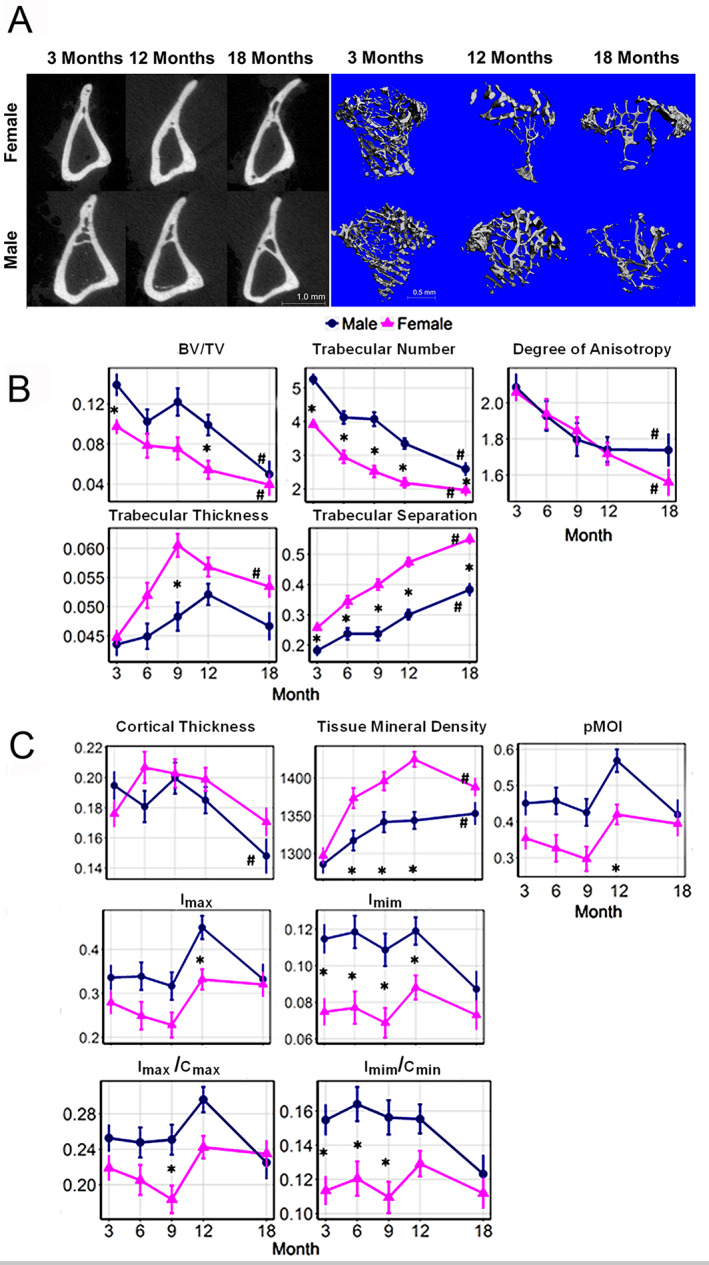
Organ‐level structural sex‐ and age‐associated changes of the skeleton. Tibias were harvested from male and female mice at 3, 6, 9, 12, and 18 months of age. Bone parameters were analyzed by microCT (*n* = 4–10 bones per sex per age). (*A*) Representative renderings of the microCT ROI from male and female mice at 3, 12, and 18 months. (*B*) Analysis of trabecular parameters: bone volume fraction (BV/TV), trabecular thickness (Tb.Th, mm), degree of anisotropy (DA), trabecular number (Tb.N, 1/mm), trabecular separation (Tb.Sp, mm). (*C*) Analysis of cortical parameters: cortical thickness (Ct.Th, mm), tissue mineral density (TMD, mg hydroxyapatite/cm^3^), polar moments of inertia (pMOI, mm^4^), maximum (I_max_, mm^4^) and minimum (I_min_, mm^4^) moments of inertia, maximum (I _max_/C_max_, mm^3^) and minimum (I_min_/C_min_, mm^3^) section moduli. *Significant difference between male and female at given month. ^#^Significant difference between months 3 and 18 for a given sex.

In the final series of studies, the transcriptomic sex‐specific differences are placed in context of the age‐associated changes in the structure and mineralization of the appendicular and axial skeletal elements over the 18‐month period in male and female mice. The results for the appendicular skeleton are presented in Fig. 8 and completely summarized in Supplemental Table S6. Bone density, quantified by bone volume fraction (BV/TV), and bone size, quantified by polar moments of inertia (pMOI), I_max_, and I_min_, were all larger in males than females. Both males and females showed an overall loss in bone density after sexual maturity is reached at approximately 3 months of age. However, across all ages, female bones tended to contain thicker but fewer and more sparsely interspersed trabeculae and to display higher tissue mineralization but smaller overall bone size compared with male bones. Many of these features were also temporally discordant between the males and females: for example, in females, the peak values of cortical thickness, trabecular thickness, and tissue mineral density were reached at 6, 9, and 12 months, respectively, whereas in males, the peak values occurred 3 to 6 months later. Results for the trabecular‐rich axial skeleton were similar to those for the tibia (Supplemental Fig. S4).

## Discussion

### Sex‐specific expression of connective tissue genes is a central element of sexual dimorphism of skeletal tissues

Connective tissue–associated genes were ~40% of the group of most significant differentially expressed genes between male and female bones. Because of the central roles that connective tissues play in all facets of the structure and function of bone, the quantitative differences in the levels of expression of these genes alone may be both a manifestation of and causal in the difference in overall skeletal size between males and females. The overlapping expression of many of these connective tissue genes in vascular tissues could also be mechanistically related to the overall differences in male and female size since at a systemic level, the extent of vascularization is related to the growth and size that a tissue can attain^(^ ^35^ ^)^ and since these two tissues share common morphogenetic developmental regulation though BMPs.^(^ ^36^ ^)^ The overlap in sex‐specific expression between skeletal and vascular tissues is consistent with our prior studies of fracture healing that showed coordinated gene expression between vascular and bone tissue formation within callus tissues,^(^ ^36^ ^)^ as well as studies showing that coupling between osteoblasts and CD31^hi^EMCN^hi^ vascular endothelium was essential for bone healing.^(^ ^37^ ^)^ Regulation of matrix mineralization and vascularization by VEGF appears to occur through distinct mechanisms in males versus females, which may also lead to the divergent physical bone traits of males and females.^(^ ^38^ ^)^ Finally, our survey of the GEO showed that a subset of genes with downregulated male‐specific differential expression in bone demonstrated coordinate downregulation in female vascular tissue in response to hyperphosphatemia in abdominal aorta but showed upregulated response as in female aortas and in bone tissue in response to mechanical loading. Two separate conclusions may be drawn from these data. First, the regulatory mechanisms that mediate the sex‐specific expression of these genes in bone show cross‐functional regulation in vascular tissues. Second, different external stimuli such as serum phosphate levels and mechanical stimulation involve regulatory mechanisms that overlap with those resulting in sexually dimorphic expression of these genes in bone.

### Morphogenetic patterning is temporally earlier, while coupled remodeling is quantitatively higher in females during their period of fecundity

Our temporal transcriptomic data showed gene‐level linkage between two sets of gene ontologies that affect bone quality (bone mineral content and morphology) and/or metabolic skeletal functions (remodeling and skeletal cell functions) and specific patterns of temporal expression. The group of gene ontologies associated with morphogenesis show elevated expression in females at earlier times than in males. The other group of ontologies associated with mineral metabolism, osteoblasts, and osteoclasts had higher expression in females concurrent with the period of female fecundity. These observations suggest two conclusions about the underlying regulatory mechanisms controlling sexual dimorphism of skeletal tissues. First, while morphometric data have all shown that in human and mice, development and aging of the axial and appendicular skeleton is dimorphic,^(^ ^1^, ^2^, ^39^ ^)^ the data presented here suggest that the timing of their postnatal development is mechanistically coordinated through the expression of a large group of genes. Such coordinated expression further suggests that the mediating mechanism(s) must be systemic because they transcend variant embryonic development and/or embryonic tissue origins that are associated with the axial and appendicular development. Second, females are more metabolically active than males and this high rate of coupled remodeling is associated with the period when they are sexually reproductive. An interesting paradox to these data is the much higher levels of gene expression in the females than in males, even though males accrue an overall higher mass of bone than females. This would suggest that the overall rate of extracellular matrix accumulation is not simply a function of the levels of gene activity but under some forms of posttranscriptional control.

Our conclusions related to an overall higher rate of coupled remodeling are all strongly supported by the histological, cell culture, and bone tissue analysis. The analysis of specific bone mRNA expression and osteoclast mRNA expression using qRT‐PCR further validated the inferred conclusions made from the transcriptomic analysis. The females also exhibited fewer but thicker trabeculae, indicative of more rounds of coupled remodeling that leads to loss of the smallest trabeculae but thickening of the remaining larger ones.^(^ ^16^ ^)^ Thus, even though female mice do not undergo menopause, the higher bone remodeling activity in the females until the end of their period of fecundity modestly compensates for a constant rate of bone loss found in both sexes after reaching skeletal maturity.

### Cortical and intramedullary populations of osteogenic cells appear to be functionally distinct

Cortical and intramedullary bone compartments arise and develop during embryogenesis and then during postnatal growth by very different mechanisms.^(^ ^40^ ^)^ In mature animals, these bone compartments also have different functional capacities to contribute to bone repair.^(^ ^41^, ^42^ ^)^ A recent study comparing skeletogenic stem cells from the intramedullary space and periosteal surfaces provided further evidence that bone contained separate populations of skeletogenic stem cells with distinct physiologic functions.^(^ ^31^ ^)^ This study showed that periosteal cell populations were defined by their unilineage potential to form only bone, their plasticity to form cartilage and bone in response to bone trauma, and their inability to support hematopoiesis. In contrast, the intramedullary population was defined by its ability to support hematopoiesis and its inherent osteogenic/chondrogenic potential.^(^ ^33^ ^)^ Our observed differences in transcriptomes of male and females that define skeletal sexual dimorphism would further suggest that there are developmental differences in skeletogenic stem cell populations that contribute to sexual dimorphism. This conclusion is based both on differences in periosteal thickness and on the higher expression of genes associated with maintenance of stem cell pluripotency in the periosteum of females at earlier time points than males. The finding that the stem cell maintenance gene *Nanog* is also preferentially expressed in the periosteal skeletal stem cell population^(^ ^31^ ^)^ and our observation of its earlier peak expression in females compared with males provides further evidence consistent with the conclusion that there is sexual dimorphism in the functionally different osteogenic cell populations. Finally, such findings of sexual dimorphism in the two separate populations are supported by both the unique quantitative and temporal transcriptomic differences that are associated with the earlier cessation of appositional growth in females over males in contrast to quantitatively higher levels of gene expression associated with remodeling found in females over males, which largely reflects intramedullary bone cell activity.

### Strengths and limitations

The primary strength of this study was the use of multiple approaches to assess the sex‐specific differences in transcriptomic expression of bone tissues. Using a basic statistical approach to determine the sex‐specific expression differences characterizes the most significant sex‐associated differences in gene expression within bone tissues independent of age. The openSESAME program was used to determine if bone tissue–specific group of differentially expressed genes was also expressed in other tissues and under what experimental or biological conditions they were expressed. Finally, using the entropy penalized expectation maximization (EPEM) algorithm modeled the sex‐specific temporal differences in transcriptomic expression of the bone tissues as male and female animals develop and age, thereby identifying sexually dimorphic biological processes that affect the development and aging processes of bone tissue.

This study also had a number of tradeoffs and technical limitations. The use of whole bone organ RNA inclusive of the marrow and periosteum for our transcriptomic analysis does not examine the gene expression in specific cell types. Thus, the specific cell types that express specific genes and the biological processes that are regulated by age and sex in a given cell type can only be inferred. While cortical or reamed trabecular bone cleaned of adherent tissues would have enabled us to examine changes more specifically in osteoblast and osteocyte gene expression, our study would have been limited to these cell types. Therefore, it is our opinion that whole organ assessments of bulk tissue RNA inclusive of the marrow and periosteum, which are informative of osteoclasts and periosteal cell gene expression, have an overall value to understanding age‐ and sex‐related changes within the bone tissue, since these cells are primarily related to remodeling and appositional modeling, growth, and response to loading.^(^ ^43^ ^)^ Other information that was gained from the analysis of the marrow was inclusive of quantifiable changes in inflammatory and immunological expression profiles that are found in both the quantitative and temporal shifts in gene expression for ontologies associated with “inflama‐aging,” which has been put forth as a major driver to systemic aging.

A second set of limitations for this study are related to how we performed our array data's statistical analysis. At the time that these studies were carried out in 2011 to 2015, our core facility did not have the pipeline set up to apply the moderated “limma” approach, which is now the standard for correcting for batch effects found in microarray data. We, furthermore, used a basic *t* test of the unlogged expression values without correcting for time to assess the male to female DE. As a response to these technical deficiencies, we have performed a comparative analysis using both moderated and non‐moderated approaches, as well as comparing the *t* test to ANOVA methods that are summarized in Supplemental Table S7. This analysis showed that the DE we used for our assessment had a much higher FDRq than the moderated ANOVA values, as well as underestimated the complement of expressed genes of the bone transcriptome that would have shown sexually dimorphic expression (704) at the 0.1 level and 398 genes at the 0.05 levels. Nevertheless, the genes we identified overlap with those found with the moderated ANOVA at least at the 0.1 level, so they represent a valid data set of sex‐associated expression differences.

A final limitation of our study was the use of a single inbred strain of mouse to carry out this study. On the one hand, the strain we used for this study is the most common one used to generate transgenic variants for most research studies. It should also be noted that comparisons across all of the inbred strains show similar sexual dimorphism in numerous skeletal structural features such as overall cortical cross‐sectional area and cortical thickness and broadly similar differences in trabecular features.^(^ ^44^ ^)^ On the other hand, there are strain‐specific variations in many of these same skeletal structural features, rates of bone growth, and age‐dependent bone loss with these differences found in both sexes.^(^ ^11^ ^)^ Other comparative studies between strains show different susceptibility to the development of obesity and diabetes.^(^ ^45^ ^)^ In this regard, future studies of strain‐specific differences in sexual dimorphic aging and disease pathogenesis will be quite informative to the specific genetic variants that interact with sex. Finally, it should be noted that the biological processes that we have inferred from our gene expression in mice related to sexual dimorphisms in bone, earlier cessation in appositional growth, and higher coupled remodeling during period active sexual reproduction are consistent with human features of bone sexual dimorphism.^(^ ^17^ ^)^

These data in general show that the timing of expression of many different biological processes is sexually dimorphic and would affect the different paces of tissue aging and the development of skeletal pathologies in males and in females. More specifically, this report provides evidence that skeletal sexual dimorphism arises through sex‐specific, temporally different processes controlling morphometric growth and coupled remodeling. The data lend credence to the idea that these processes are controlled by functionally distinct skeletogenic populations of cells. Finally, the data presented here illustrate the power of using time as a dynamic factor in transcriptomic analysis to leverage underlying transcriptomic features that are informative of biological processes.

## Disclosures

All authors state that they have no conflicts of interest.

### Peer Review

The peer review history for this article is available at https://publons.com/publon/10.1002/jbm4.10579.

## Supporting information


**Appendix** **S1**. Supplementary.

**Table S1.** Summary of the Technical History of the RNA Preparations and Microarray Analysis
Sheet one contains the technical history of the preparation of the RNA including the age, sex, and genotype of the mouse, the mouse ID applied to all further specimens and data points from that animal, the DOB of mouse date of RNA isolation, individual who isolated the RNA, RIN value of the sample, and any technical notes related to the sample. Sheet two gives concentration values for each RNA sample. Sheet three contains the cDNA concentrations.

**Table S2.** Sex Differential Summary of the 353 Genes Identified by *t* Tests of Male Versus Female, Across All Samples Without Correcting for Time Point Non‐Log Transformed Values up to FDR q Threshold at 0.025
Gene entrez ID, gene symbol, gene description *t* test value, and FDRq value are included.

**Table S3.** Summary of Coordinate Rodent Series From the SESAME Analysis (as Described in the Text)

**Table S4.** Complete Data Set for Gene Expression Data Used for EPEM Algorithm Cluster Tables
Sheet one contains mean values across all time and unlogged values for male and female groups and the unlogged expression ratio between males and females. Sheet two contains the male‐to‐female cluster comparisons aligned to the cross tabulation between clusters. Gene entrez ID, gene symbol, and gene description are included with mean age sex group values. Sheet 2 and sheet 3 contain the concordant and discordant genes groups. Gene entrez ID, gene symbol, mean age and sex group values, mean values across all time and unlogged values for male and females groups, and the unlogged expression ratio between males and females are presented. Sheet 4 contains skeletal‐associated gene ontologies used in the manual annotation of the discordant and concordant gene sets used for secondary ontology assessments.

**Table S5.** **A**. Ontology Groups Used for Targeted Assessments in Fig. 6 (as Described in the Text)
**Table S5B**. Complete Data Set for Gene Expression Data Used for Selected Cluster Comparisons in Fig. 6*A–C*)
Top headers are for manual curation of the different gene sets in Fig. 6. Gene entrez ID, gene symbol, gene description and cluster comparisons aligned to the cross tabulation between clusters, mean age sex and group values, mean values across all time, unlogged values for male and female groups, and the unlogged expression ratio between males and females are presented.

**Table S6.** MicroCT Data Means by Sex and Age
Sheets 1 to 3 are the individual microCT values for tibia trabecular, vertebrae trabecular, and tibia cortical parameter, respectively. Specimen ID, age, sex, bone, and data features are denoted in each sheet.
