# Supplementary material for: Temporal and Quantitative Transcriptomic Differences Define Sexual Dimorphism in Murine Postnatal Bone Aging
Source: JBMR Plus. 2021 Dec 10;6(2):e10579. doi: 10.1002/jbm4.10579 (PMC8861981; doi:10.1002/jbm4.10579)
Supplement: Supplementary file 1 — Appendix S1. Supplementary. [file JBM4-6-e10579-s002.docx]

# **Supplementary Materials**

1) **Supplemental Excel Table 1 Summary of the technical history of the RNA preparations and microarray analysis.**

Sheet one contains the technical history of the preparation of the RNA including the age sex and genotype of the mouse, the mouse ID applied to all further specimens and data points from that animal, the DOB of mouse date of RNA isolation, individual that isolated the RNA, RIN value of the sample and any technical notes related to the sample. Sheet two is concentrations values for each RNA sample. Sheet three the cDNA concentrations.

2) **Supplemental Excel Table 2 Sex Differential** Summary of the 353 Genes identified by T tests of Male vs Female, Across All Samples without Correcting for Timepoint Non Log Transformed Values up to FDR q Threshold at .025. Gene Entrez ID, Gene Symbol, Gene description T-Test value and FDRq value are included.

3) **Supplemental Table 3 Summary of coordinate rodent series from the SESAME analysis** (As described in the text below)

4) **Supplemental Excel Table 4 Complete Data Set for Gene Expression Data Used for EPEM Algorithm Cluster Tables.** Sheet one contains Mean values across all time and unlogged values for male and females groups and the unlogged expression ratio between males and female. Sheet two contains the male to female cluster comparisons aligned to the cross tabulation between clusters. Gene Entrez ID, Gene Symbol, Gene description are included with mean age sex group values. Sheet 2 and Sheet 3 contain the Concordant and discordant genes groups. Gene Entrez ID, Gene Symbol, mean age and sex group values, Mean values across all time and unlogged values for male and females groups and the unlogged expression ratio between males and females is presented. Sheet 4 contains skeletal associated gene ontologies used in the manual annotation of the discordant and concordant gene sets used for secondary ontology assessments.

**5) Supplemental Table 5A Ontology Groups used for Targeted Assessments Figure 5** (as described in the text)

6)**Supplemental Table 5B Complete Data Set for Gene Expression Data Used for Selected Cluster Comparisons in Figure 5 A-C)**Top Headers are for Manual Curation of the Different Gene Sets in figure 5. Gene Entrez ID, Gene Symbol, Gene description and cluster comparisons aligned to the cross tabulation between clusters, mean age sex and group values, Mean values across all time and unlogged values for male and females groups and the unlogged expression ratio between males and females is presented

6) **Supplemental Excel Table 6 MicroCT Data Means By Sex and Age** Sheets 1-3 Respectively are the individual microCT values for tibia trabecular, vertebrae trabecular and tibia cortical parameter.

Specimen ID age sex bone and data features are denoted in each sheet.

7) Figure S1 Principal Component Analysis of Microarray Batches Compared to Age of Mice of Mice

8) Figure 2S OpenSESAME Validation Study Coordinate sex-specific gene signature of bone associates with te gene expression changes seen in fetal Liver knock out study.

9) Figure 3S Comparative Biological Ontology Assessment of the Quantitative Set of DE to the Temporal Gene Groupings.

10) Figure 4S: Comparison of Trabecular bone structure in the tibia and vertebra of male and female mice from 3 to 18 months of age:

**A. RNA Processing and Quality Controls**

*1) RNA Processing and Microarray Development:* The bone RNAs used in this project were all prepared from humeri of an aging C57BL/6 wild type mice and heterozygous mice in which the Brd2 gene had been functionally deleted. An additional set of 4 RNAs were prepared from 3 month old female C57BL/6 wild type mice purchased from Jackson Laboratories, Bar Harbor, ME. A total of 80 RNA samples equally composed of male and female mice from the wild type and mutant mice and the additional four female mouse samples were processed and scanned separately in two batches, which were balanced for all experimental variables. 200ng of RNA from each bone mRNA sample was labeled with biotin using the Ambion WT Expression Kit (Life Technologies, Grand Island, NY) according to the manufacturer's protocol, followed by the GeneChip WT Terminal Labeling and Controls Kit (Thermo Fisher, Waltham, MA). Labeled, fragmented DNA was hybridized to the Affymetrix Mouse Gene 1.0 ST Array for 18 hours in a GeneChip Hybridization oven 640 at 45°C with rotation (60 rpm). Hybridized samples were washed and stained using an Affymetrix fluidics station 450, and after staining, microarrays were immediately scanned using an Affymetrix GeneArray Scanner 3000 7G Plus. Supplemental Table 1 is a summary of the technical history of the RNA preparations and microarray cDNA preparations.

*2) Microarray Processing and Quality Control Assessments* All 84 CEL files were normalized together to produce gene-level expression values using the implementation of the Robust Multi-Array Analysis (RMA) algorithm^29^ in the affy R package (version 1.36.1) and an Entrez Gene-specific probe set mapping (version 14.0.0) from the Molecular and Behavioral Neuroscience Institute (Brainarray) at the University of Michigan. Array quality was assessed by computing Relative Log Expression (RLE) and Normalized Unscaled Standard Error (NUSE) values using the affyPLM R package (version 1.34.0), and by re-normalizing the CEL files using Expression Console (build 1.3.0.187) to generate the Area Under the [Receiver Operating Characteristics] Curve (AUC) metric using positive and negative control probes. All samples had similar distributions of RLE and NUSE values, and AUC values greater than 0.85, indicating that all samples were of suitable quality for analysis. Expression values were processed using the implementation of ComBat in the sva R package (version 3.4.0) to adjust for any technical effects with respect to microarray processing. All microarray analyses were performed using the R environment for statistical computing (version 2.12.0). Raw CEL files and RMA-normalized gene expression values have been deposited in the Gene Expression Omnibus (GSE141451). Differences between gene expression between male and female control groups were examined using Student's t test, assuming equal variance, across all timepoints, with correction for multiple hypothesis testing accomplished using Storey's positive false discovery rate (pFDR)^30^ in SAS (version 9.4, SAS Inc., Cary, NC).

*3 Quality Control Assessments*

*a) Sex Specific Gene Expression:* The sex of the animals was confirmed using the expression of several constitutively expressed Y-linked genes (*Ddx3y*, *Eif2s3y*, *Kdm5d*, and *Uty*) and the female-specific marker gene *Xist*.

*b) Normalization and Quality Control Assessments of Microarray Batches:* The arrays were normalized using the Robust Multiarray Average (RMA) algorithm and a library file that maps the probes on the array to unique Entrez Gene IDs. The expression values were log2-transformed by default. The quality of the microarrays was further assessed using two objective QC metrics (not shown). In batch 1, there was one sample that did not pass either of these quality filters: sample 259 (6 month female heterozygote). There was a second sample that was very nearly considered an outlier by these metrics: sample 282 (12 month male wild-type). There were however no technical problems associated with either of these samples during the microarray procedures and they were included in the analysis. Expression values were further verified based on sex specificity and Brd2 expression which was half to 2/3 of normal in the heterozygote mice.

Principal Component Analysis (PCA) was then used to collapse the variance between samples into a smaller set of orthogonal Principal Components (PCs). While this analysis showed that there was no apparent batch effect due to the microarray both plots showed large difference in global gene expression between two groups of samples corresponding to the 0/3/6-month and 9/12/18-month groups. In batch 1 two outliers 259 and 282 clustered to the extreme of their clusters (0/3/6-month and 9/12/18-month, respectively), suggesting that they do in fact represent outliers. In batch 2, there are two samples (sample 236, 12 month male wild-type; sample 103, 18 month male wild-type) that unexpectedly clustered with the 0/3/6 month’s group rather than the 9/12/18 group. These variance do not look like a case of switched samples however because there are no 0/3/6 month samples (in either batch) that cluster with the 9/12/18 month samples. The one discernable source of technical variation (e.g., sample processing) between the time points was based on the individual that processed the 0/3/6 and 9/12/18 month groups however no other source of variation in methodology used to prepare the samples can be ascribed to the differences in the time points.

**
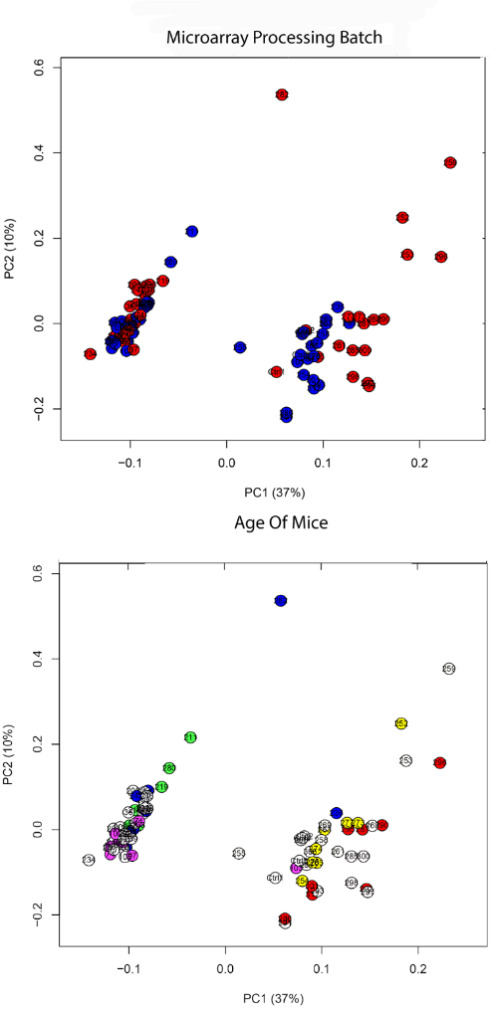
**

**Supplemental Figure 1** PCA Analysis. Upper Panel Analysis by Microarray processing Batch. Red= Batch one Blue = Batch2.Lower Panel is by Age of Mice Genotype Groups are denoted in the lighter colors. Age groups are denoted Red = 3 months, Yellow =6 months, Green =9 months, Blue=12 months and Purple =18 months. Individual animal numbers are denoted within each Microarray Circle .

*c) Re-assessments of DE Using Multiple Statistical Methods* At that time these experiments were initially carried out in 2011 -2012 we used the state of the art methods to normalize microarray expression values (Johnson WE, Li C, Rabinovic A. Adjusting batch effects in microarray expression data using empirical bayes methods. Biostatistics. 2007;8(1):118–27.) At that time however the core facility did not have the pipe line set up to apply limma for the downstream statistical assessments of determining differential expression (DE) and further analysis of the normalized values used much simpler methods. In recognition of the more common use of moderated assessments methods for data analysis we have compared multiple statistical approaches including the moderated (limma) approaches to determine if this will change the overall selection of the sex DE. We include these additional analysis to provide a more complete assessment of all the various ways and normalization methods to assess the DE but show that in general that quality of our starting data was very good.

Using the same 37samples (excluding three that did not meet QC) that were used for the original study the following additional analysis were run.

- Ordinary two-way ANOVA, using log-transformed data
- Ordinary two-way ANOVA, using non-log-transformed data
- Moderated two-way ANOVA, using log-transformed data
- Moderated two-way ANOVA, using non-log-transformed data

Two-sample t tests across all samples (i.e., not correcting for timepoint)

- Student t test (assuming equal variance), using log-transformed data
- Student t test (assuming equal variance), using non-log-transformed data
- Moderated t test, using log-transformed data
- Moderated t test, using non-log-transformed data

Storey FDR q values using the q value function in the q value R package (rather than Benjamini-Hochberg FDR q values) were used, as this was how the analysis presented in the paper was done. The values that were produced were almost the same as those produced by SAS although slightly larger numbers of genes were identified for the matched group to that which we determined using SAS. For the FDR q values across all genes only one gene Spink1 (Entrez Gene ID 20730) was left out of the tables in the original excel files.

Table 7A below enumerates how many genes were significant for each approach at various Storey FDR q thresholds. There was not a huge difference between the moderated and ordinary approaches for the simple T test groups, so we believe this is sufficient evidence that limma (moderated approach) doesn’t make a difference. Finally, there were only two genes in the DE signature as well that were not significant in the moderated t test (using non-log-transformed data): Table 7B Both genes were expressed at very low levels in all samples (about the same as or lower than Y-linked genes in female mice, i.e., absent) and so they appear to have been false positives in the original signature. It should be noted that the small subset of genes and the higher FDRq for our DE was potentially due to our use of Student T test assessment and use of non-log-transformed values which was dictated the criteria we set for our control experiments using open SESAME in which we used non-log-transformed values to set the baseline.

| **Table 7A**  **Assessing significance of sex after correcting for timepoint (two-way ANOVA)** | | | |  |
| --- | --- | --- | --- | --- |
|  | Non-log-transformed expression | | Log-transformed expression | |
| FDR q threshold | Ordinary  ANOVA | Moderated  ANOVA | Ordinary  ANOVA | Moderated  ANOVA |
| 0.25 | 2364 | 2173 | 2573 | 2653 |
| 0.2 | 1790 | 1672 | 1939 | 1998 |
| 0.1 | 687 | 706 | 783 | 839 |
| 0.05 | 335 | 350 | 364 | 398 |
| 0.01 | 96 | 102 | 110 | 118 |
|  |  |  |  |  |
| **t tests, male vs female, across all samples without correcting for timepoint** | | | |  |
|  | Non-log-transformed expression | | Log-transformed expression | |
| FDR q threshold | Student  t test | Moderated  t test | Student  t test | Moderated  t test |
| 0.25 | 358 | 353 | 430 | 433 |
| 0.2 | 259 | 275 | 294 | 288 |
| 0.1 | 114 | 128 | 147 | 151 |
| 0.05 | 66 | 69 | 86 | 102 |
| 0.01 | 35 | 34 | 41 | 49 |

**Table 7B**

| Entrez Gene ID | Symbol | Student FDR q | Moderated FDR q |
| --- | --- | --- | --- |
| 723858 | Mir379 | 0.181782125 | 0.66527753 |
| 791275 | Gm9885 | 0.10730678 | 0.25501538 |

While the best approach to have collected the sex linked DE would have been the moderated two-way ANOVA performed using the log-transformed data this approach produces a much larger number DE genes (704) at the 0.1 level and 398 genes at the 0.05 levels. We also want to note using non-moderated non log transformed values increases the inherent variability of the data used for statistical assessments thus increased FDR of the selected group of DE. However we want to note that the DE genes of the non-log transformed T test format all exist within704 genes seen for the moderated ANOVA. So ideally while DE at the FDR level of 0.1 would be the best for the assessment of the DE it makes little difference to data presented as to what the main gene ontology functions are in the sex specific DE are.

**B.** **openSESAME Analysis**

Four Y-linked genes (*Uty*, *Kdm5d*, *Eif2s3y*, and *Ddx3y*) and the X-linked lncRNA *Xist* were excluded from the openSESAME analysis as they are constitutively only expressed in either male or female tissues, respectively, and their inclusion leads to a biased determination. Student's *t* test (assuming equal variance) was carried out on the entire transcriptomic data set after excluding these five genes, and three groups of genes with varying levels of statistical significance in differential expression between sexes (FDR *q* < 0.05, 0.1 and 0.20) were used in these analyses. These three sex-specific gene groups were respectively composed of 27/34, 51/60 and 106/148 up/down-regulated genes in male relative to female mice. All publicly available CEL files from GEO as of October, 2014 in which RNA had been profiled on Affymetrix GeneChip microarrays were downloaded for use in the openSESAME query. The openSESAME query was then performed using the three groups of differentially regulated genes. This query identified 0, 81, and 2,600 datasets (termed "Series" in the GEO nomenclature) from the queries with the smallest, mid-sized, and largest gene signatures, respectively.

Eighty-one GEO Series were obtained from the analysis using the middle size gene set with FDR values of 0.10 as the query genes. The analysis identified 81 GEO Series that exhibited significant coordinate expression of the sex-specific bone signature. These 81 series were composed of 26 mouse, 2 rat, 52 human and one rhesus study. We focused our further investigation on the 28 rodent studies (**Supplemental Table 2**). Data included in the table include the significance rank within the 81 series, the experimental species, the GEO Series ID, whether the sex of the sample from which the profiled bio specimens were obtained was noted or could be determined, the tissue type and the experimental perturbation or biological variables being examined in the study.

**Supplemental Table 3 Open SEASME Summary of Coordinate Rodent Series**

| **Significance Ranking** | **GEO #** | **Species** | **Both sexes?** | **Tissue Type** | **Perturbation** |
| --- | --- | --- | --- | --- | --- |
| 21 | GSE22286 | Rattus norvegicus | No | Bone | Forelimb loading |
| 64 | GSE4242 | Rattus norvegicus | Possibly | Cultured cortical neurons from embryos | Nicotine exposure |
| 8 | GSE10246 | Mus musculus | Yes | Huge variety | None |
| 10 | GSE20465 | Mus musculus | No | Breast, liver, spleen, blood cell, thymus | Breast cancer |
| 18 | GSE15998 | Mus musculus | Yes | Huge variety | None |
| 22 | GSE11291 | Mus musculus | No | Neocortex, muscle, heart | Diet |
| 29 | GSE27245 | Mus musculus | Possibly | Neurons derived from embryos | Top2beta knockout, different treatments, different stages of differentiation |
| 31 | GSE41203 | Mus musculus | No | Islets of Langerhans | Diabetes |
| 32 | GSE41342 | Mus musculus | No | Bone, cartilage | DMM surgery vs. control surgery vs. no surgery |
| 36 | GSE40022 | Mus musculus | Unclear | Bone marrow | PML-RARA expressing vs. wild type |
| 41 | GSE35592 | Mus musculus | Yes | Fetal liver cells | Klf3 KO vs. WT, TER119+ or - |
| 42 | GSE8512 | Mus musculus | Yes | Bone marrow macrophages | ApoE deficiency |
| 46 | GSE18567 | Mus musculus | Unclear | Cochlear tissue | A9 knockout mice vs. wild type at P3, P7, P13, P60 |
| 47 | GSE44647 | Mus musculus | Unclear | Lung, spleen, and liver | 9V null vs. wild type mice |
| 50 | GSE2278 | Mus musculus | No | Spleen, leukocytes | Trauma response |
| 51 | GSE10744 | Mus musculus | No | Lung, kidney, brain, heart, testis, liver | Males of different strains |
| 52 | GSE38120 | Mus musculus | Yes | Aortic vascular cells | Strain and diet |
| 54 | GSE33341-mouse4302 | Mus musculus | Yes | Blood | Staph and e. coli infection vs. healthy mice |
| 56 | GSE17160 | Mus musculus | Unclear | Fibroblasts | WT and TSG6-KO mice with different treatments |
| 57 | GSE32681 | Mus musculus | No | Lacrimal gland, salivary gland | LTBR-Ig treatment |
| 60 | GSE20524 | Mus musculus | No | Blood | Candida and staph infections vs. healthy mice |
| 61 | GSE27397 | Mus musculus | Unclear | Unclear | C57BL/6 vs. 129S2/SvPas |
| 62 | GSE57818 | Mus musculus | No | Thoracic and abdominal aorta | High phosphate vs. standard chow diet |
| 63 | GSE5130 | Mus musculus | Unclear | T and B cells | None |
| 71 | GSE4288 | Mus musculus | Unclear | Macrophage cell line | Cell vs. polysome vs. mRNP |
| 72 | GSE1025 | Mus musculus | No | Leg muscle | Dystrophin deficiency at different ages |
| 73 | GSE40531 | Mus musculus | Unclear | Bone marrow, spleen, and thymus | Mouse models of AML |
| 77 | GSE17933 | Mus musculus | No | Lung | Exposure to chemicals |

An internal validation of the Gene set selection for the sex related nature of the 111 genes that were used to screen GEO data base is next presented. In this experiment, the mouse series GSE35592 was chosen for its high level of association (Fisher *p* = 0.0002, FDR *q* = 0.0511) with the differential gene set of mouse bone. The data descriptor that had been provided in the GEO was, “The aim of this experiment was to investigate the role of KLF3 in regulating gene expression at different stages throughout the erythroid maturation process. Affymetrix microarrays were performed on fetal liver cells (both TER119- progenitor cells and TER119+ erythroblast cells) from E14.5 wildtype and Klf3 KO mice.” Upon downloading this data set we used the expression values of the five sex-specific genes that were excluded from our search list (Uty, Kdm5d, Eif2s3y, Ddx3y and Xist) to appropriately identify the sex of the mice that had been assessed in this microarray study. The coordinate group of genes that was differentially expressed in bone showed strong association to the Ter expression pattern in this experiment, but more strikingly a subset coordinate group showed an association that was able to accurately identify the sex of mice in this study (**Supplemental Figure 4)**.


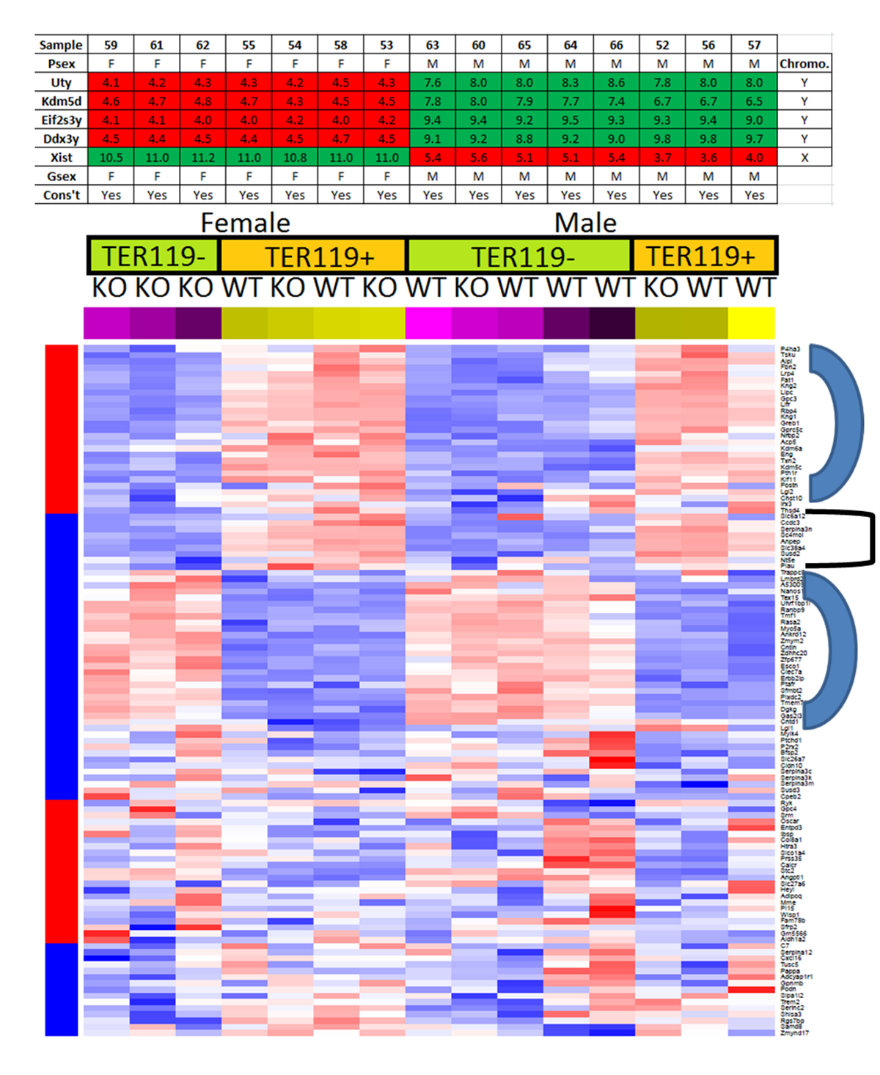


**Supplemental Figure 2.**OpenSESAME Validation Study Coordinate sex-specific gene signature of bone associates with the gene expression changes seen in fetal Liver knock out study. Experimental conditions are shown across the top of the heat maps. The red and green table of sex specific genes at the top of the figure show the unlogged expression values from which the base line of background expression of the control experiment was set. In the Heat map red denotes increased and blue decreased expression from values seen for the sex differential gene expression seen in female bone tissues. The side red and blue bars indicates the expression of these genes groups in the published experiment. The small group of genes that moved in the opposite direction from the published data map to the sex of mice.

**Table 5A Ontologies used to Construct Figures 4**

Morphological Dimorphism

| Figure Panel |  | Cluster * | Canonical pathway or diseases and functions(fx) | IPA ontology |
| --- | --- | --- | --- | --- |
| Stem Cell |  | M2/F7 | Pathway | Human embryonic stem cell pluripotency |
| Morphology 1 |  | M2/F3 | Disease and fx | Morphology of vertebrae  Abnormal morphology of vertebrae  Abnormal morphology of limb bone |
| Morphology 2 |  | Male increasing female flat  M1&2/F4 | Diseases and fx | Development of axial skeleton  Fusion of bone  Fusion of atlas  Fusion of occipital bone  Fusion of vertebrae |
| Morphology 3 |  | Male increasing female flat  M1&2/F4 | Diseases and fx | Abnormal morphology of bone |

Dimorphism in coupled remodeling

| Figure Panel |  | Cluster | Canonical pathway or diseases and functions | IPA ontology |
| --- | --- | --- | --- | --- |
| OC1 |  | M1/F1 | Diseases and fx | Osteoclastogenesis |
| Remodeling |  | Male flat female increasing  M4/F2&1 | Diseases and fx | Mineralization of bone  Mineralization of connective tissue  Quantity of bone |
| OB |  | Male increasing female flat  M4/F2&1 | Diseases and fx | Quantity of Osteoblasts  Differentiation of bone marrow mesenchymal cell-derived osteoblasts |
| OC2 |  | Male increasing female flat  M4/F2&1 | Diseases and fx | Quantity of Osteoclasts  Differentiation of Osteoclasts |

Estrogen Metabolism and Fecundity

| Figure Panel |  | Cluster | Canonical pathway or diseases and functions | IPA ontology |
| --- | --- | --- | --- | --- |
| E2 Synthesis |  | Male flat female decreasing  M7&4/F7 | Pathway | Estrogen Biosynthesis  Estrogen Biosynthesis |
| E2 Signaling |  | M/1/F1 | Pathway | Estrogen Receptor Signaling |
| E2 Signaling |  | M7/F7 | Pathway | Estrogen Receptor Signaling |
| Germ cell Quantity |  | M2/F7 | Diseases and fx | Quantity of Germ Cells  Quantity of Ovary  Quantity of Oocytes  Lack of Germ Cells |

*Denotes the specific shift from a male (M) cluster number to a Female (F) cluster number.


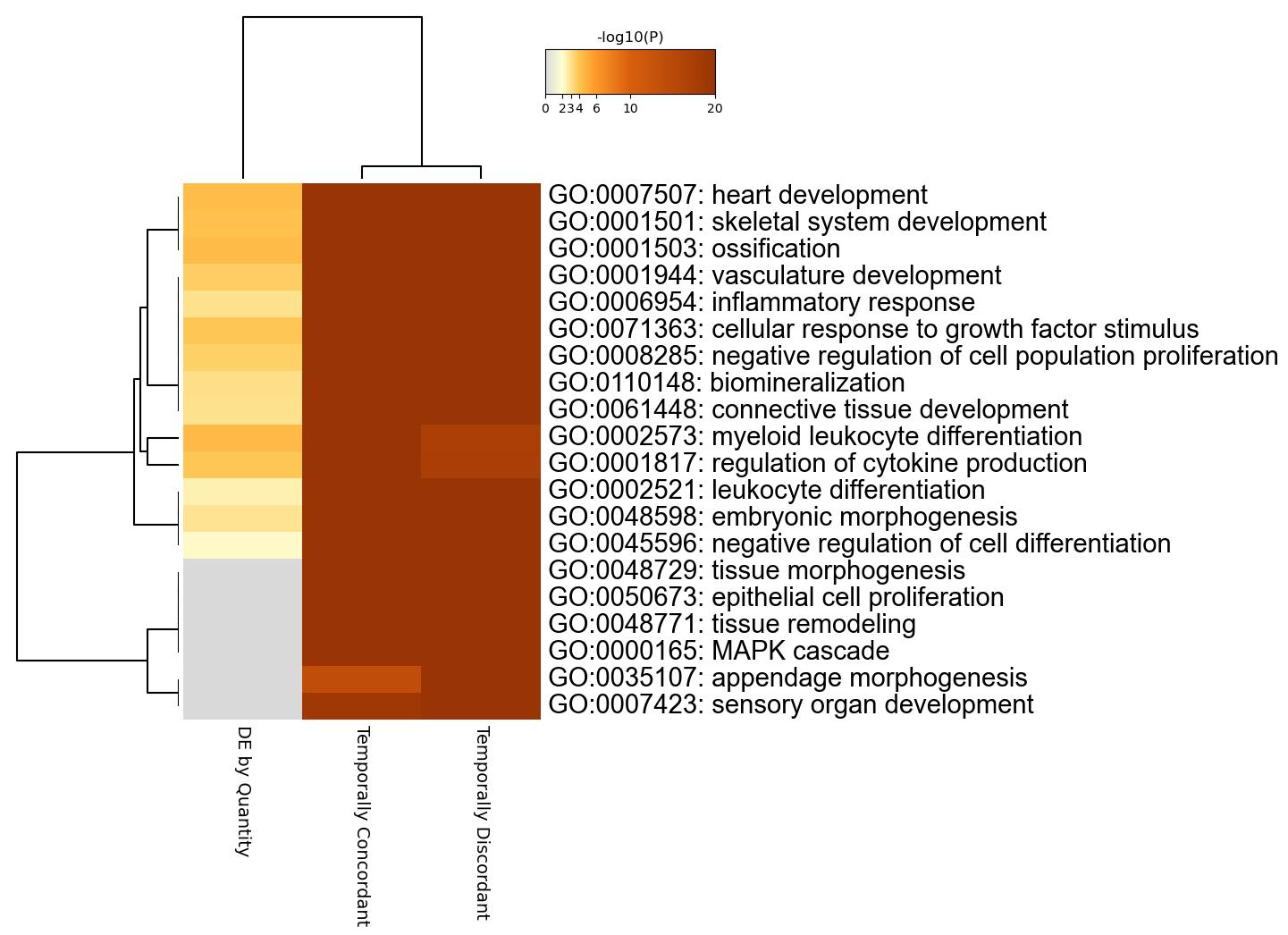


**Supplemental Figure 3** Comparative Biological Ontology Assessment of the Quantitative Set of DE to the Temporal Gene Groupings. Heatmaps are from the enrichment analysis carried out in Metascape for DE having FDRq 0.25 compared to the temporally discordant and concordant genes groups identified by IPA as skeletal tissue related. The heatmap cells are colored by their p-values as shown in the figure.

**C. Histological and Immunohistological methods:**

Cross sections were cut at approximately the same location of mid-diaphysis for all bones. Samples were excluded if there was removal of periosteum during harvesting or damage during embedding. For each analysis 6 sections were processed (3 female, 3 male) and images were taken at 20X magnification with an Olympus BX 51 microscope under bright light and low fluorescence to enhance the contrast between bone and periosteum. Periosteum thickness was defined as the perpendicular distance from the furthest border of the periosteum to the bone surface. Approximately 10 fields of view were captured to enable analysis of the entire section, and a total of 50 measurements were taken for each section. All data were analyzed using GraphPad Prism 8 software. Unpaired *t* tests were used to compare sections from different sexes. Paraffin embedding and decalcification was carried out as described above for cathepsin K immunohistological analysis. Antigen retrieval was performed flowing the manufacturer's protocol by using vector antigen unmasking solution (Vector Laboratories, Burlingame, CA 94010, Lot. ZD0327). The sections were permeabilized with 0.3% Triton X-100 in PBS and blocked with 1% BSA + 0.1% Triton X-100 in PBS one hour at RT. Sections were incubated overnight with primary antibody cathepsin K (Abcam, ab19027, 1:100 dilution) or blocking buffer as negative control at 4 °C. The sections were washed with PBS and incubated with secondary antibody Alexa 488 donkey anti-rabbit 1:500 (Invitrogen, A21206) for 1.5 hour at RT. After washing in PBS for three times, the sections were treated with True View autofluorescence quenching solution (Vector Laboratories, SP-8400) for 3 min according to supplied protocol.

**D. Detailed Methods of MicroCT Analysis**

Scans were performed on a μCT 40 system (Scanco Medical, Brüttisellen, Switzerland) with voltage, current, and integration time of 70 KVp, 114 μA, and 200 ms, respectively. Trabecular bone was analyzed in the metaphyses of the proximal tibia and L5 vertebral body. Cortical bone was analyzed in the diaphyses of the long bones. For the diaphyses, the cortical ROI was an 800μm long segment that was centered at the mid-diaphysis ~4 mm from the distal fib-fib junction. For the metaphyses of the three-month-old animals, the trabecular region of interest (ROI) extended from 40 μm to 936 μm away from the growth plate, along the long axis of the bone. For the older animals, the length of this trabecular ROI was scaled by the ratio of the average bone length for the age group to the average bone length for the three-month-olds so that the ROI size would remain anatomically proportional as the animals grew. For the vertebral body, the trabecular ROI extended from 40 μm cranial to the caudal growth plate to 40 μm caudal to the cranial growth plate. The boundary between the trabecular compartment and cortical shell was delineated with a semi-automated image-processing algorithm available from the system manufacturer (Scanco Medical). Global thresholding (22.5% of the maximum intensity) for tibia was used with thresholds determined by an iterative method (Scanco Medical). Tissue mineral density was calculated with the aid of a standard curve obtained from a scan of a hydroxyapatite phantom consisting of five different mineral densities.


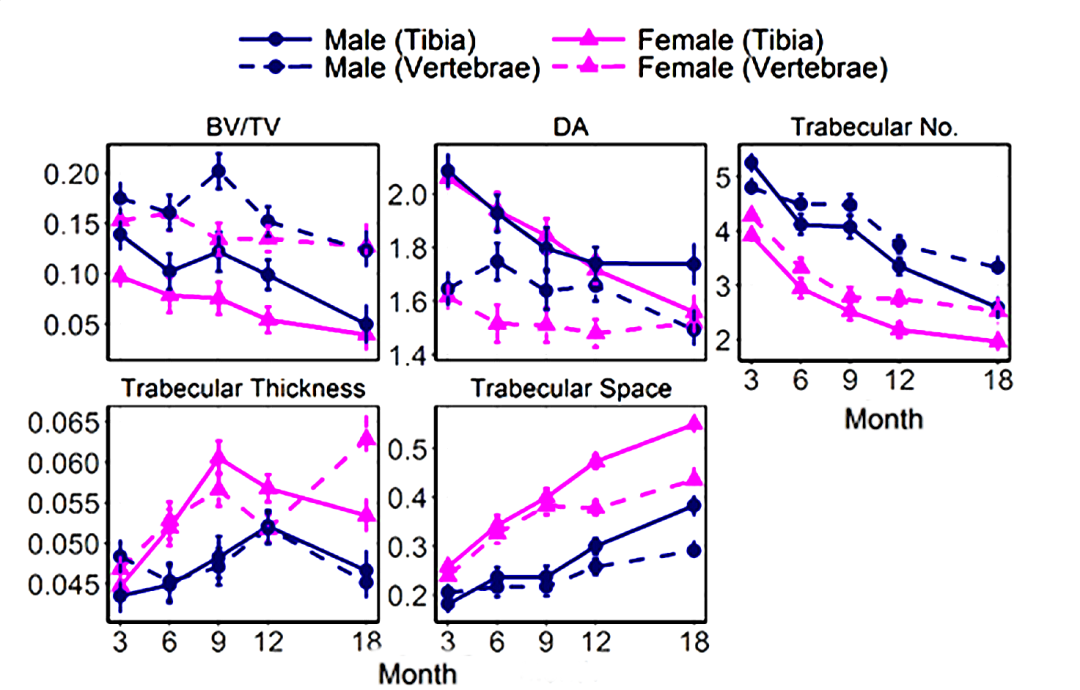


Supplemental Figure S4 : Comparison of Trabecular bone structure in the tibia and vertebra of male and female mice from 3 to 18 months of age: Vertebrae and tibiae were harvested from the same mice at 3, 6, 9 12 and 18 months of age and analyzed by micro-CT (n=4-10 tibiae, and n=4-11 vertebrae per age per sex) to quantify bone volume fraction (BV/TV), degree of anisotropy (DA), trabecular number (Tb.N), trabecular thickness (Tb.Th), and trabecular separation (Tb.Sp).
